# Immunoinformatics assisted profiling of West Nile virus proteome to determine immunodominant epitopes for the development of next-generation multi-peptide vaccine

**DOI:** 10.3389/fimmu.2024.1395870

**Published:** 2024-05-10

**Authors:** Alaa Karkashan

**Affiliations:** Department of Biological Sciences, College of Sciences, University of Jeddah, Jeddah, Saudi Arabia

**Keywords:** computer-assisted vaccine designing, reverse vaccinology, immunoinformatics, molecular docking, MD simulation

## Abstract

Emerging infectious diseases represent a significant threat to global health, with West Nile virus (WNV) being a prominent example due to its potential to cause severe neurological disorders alongside mild feverish conditions. Particularly prevalent in the continental United States, WNV has emerged as a global concern, with outbreaks indicating the urgent need for effective prophylactic measures. The current problem is that the absence of a commercial vaccine against WNV highlights a critical gap in preventive strategies against WNV. This study aims to address this gap by proposing a novel, multivalent vaccine designed using immunoinformatics approaches to elicit comprehensive humoral and cellular immune responses against WNV. The objective of the study is to provide a theoretical framework for experimental scientists to formulate of vaccine against WNV and tackle the current problem by generating an immune response inside the host. The research employs reverse vaccinology and subtractive proteomics methodologies to identify NP_041724.2 polyprotein and YP_009164950.1 truncated flavivirus polyprotein NS1 as the prime antigens. The selection process for epitopes focused on B and T-cell reactivity, antigenicity, water solubility, and non-allergenic properties, prioritizing candidates with the potential for broad immunogenicity and safety. The designed vaccine construct integrates these epitopes, connected via GPGPG linkers, and supplemented with an adjuvant with the help of another linker EAAAK, to enhance immunogenicity. Preliminary computational analyses suggest that the proposed vaccine could achieve near-universal coverage, effectively targeting approximately 99.74% of the global population, with perfect coverage in specific regions such as Sweden and Finland. Molecular docking and immune simulation studies further validate the potential efficacy of the vaccine, indicating strong binding affinity with toll-like receptor 3 (TLR-3) and promising immune response profiles, including significant antibody-mediated and cellular responses. These findings present the vaccine construct as a viable candidate for further development and testing. While the theoretical and computational results are promising, advancing from in-silico predictions to a tangible vaccine requires comprehensive laboratory validation. This next step is essential to confirm the vaccine’s efficacy and safety in eliciting an immune response against WNV. Through this study, we propose a novel approach to vaccine development against WNV and contribute to the broader field of immunoinformatics, showcasing the potential to accelerate the design of effective vaccines against emerging viral threats. The journey from hypothesis to practical solution embodies the interdisciplinary collaboration essential for modern infectious disease management and prevention strategies.

## Introduction

The West Nile virus WNV is the causative agent of vector-borne infectious illnesses primarily observed within the United States. The primary mode of transmission is predominantly by the bite of a mosquito that has been infected. The transmission of the WNV occurs when an infected mosquito transfers the disease-causing agent to an intermediate host, typically other animals. Subsequently, these infective animals can then transmit the virus to human individuals (1). Instances of WNV infections manifest during the period of mosquito activity, commencing from the summer months and extending through the autumn season. Currently, there is a lack of commercially accessible vaccines or medications for the treatment of WNV infection in human populations. Auspiciously, a majority of individuals afflicted with WNV infection do not experience symptoms of illness. Approximately 20% of persons who are infected experience the manifestation of a fever and accompanying symptoms. Moreover, it has been shown that approximately 1 out of every 150 individuals who are infected with the disease acquire a severe and potentially lethal condition (2). Furthermore, it is worth noting that a majority of individuals within various communities, approximately 80%, do not exhibit any signs of infection. However, in extreme instances, multiple symptoms may manifest, such as high-grade fever, neck stiffness, tremors, muscle weakness, eyesight loss, paralysis, and numbness (3). The process of recuperation following a severe illness might span from several weeks to many months. In certain cases, where the virus impacts the central nervous system, it may result in enduring impairments (4). Given its significant pathogenicity and the existence of effective medicine and immunization, it is imperative to implement concerted measures to address the infection (5).

Antibiotics are generally ineffective in treating viral infections due to the significant diversity and high mutation rate of viral genomes. Nonetheless, alternative treatments, including hydration and pain relievers, can offer relief (6). Vaccination offers an alternative strategy for the permanent eradication of infections. The risk of infection can be reduced through the use of appropriate insect repellent and by adopting protective measures such as wearing long-sleeved shirts and pants to decrease the chances of mosquito bites (7). The virus has been detected in several bird species, notably jays and crows, which have shown symptoms and mortality linked to the infection. Furthermore, reports suggest that birds can become infected with the virus within a week, and there is evidence of viral spread within bird populations. The genetic structure of the West Nile Virus (WNV) comprises a single-stranded RNA molecule with a positive-sense orientation (8). The primary role of the genome is twofold: firstly, to serve as the sole viral mRNA, and secondly, to act as the template for the production of the complementary minus-strand RNA (9).

The production of vaccines is a highly significant subject in the contemporary period, as it pertains to a diverse array of transmissible and infectious diseases. Additionally, vaccines can be employed as a preventive measure against certain diseases, including autoimmune conditions. Historically, the utilization of pasture vaccinology for vaccine development was characterized by its cost-effectiveness, lengthy duration, and inherent risks, occasionally resulting in the inability to successfully cultivate and propagate the desired study specimens inside laboratory settings (10).

Vaccination aims to confer protective immunity to the host organism against pathogens by eliciting both innate and acquired immune responses, which are subsequently activated upon encountering the pathogens (11). The genomes of various bacterial and viral pathogens have been made accessible in biological databases through the utilization of bioinformatics technologies. Reverse vaccinology utilizes the available databases by leveraging the sequencing data for various objectives (12). Bioinformatics, immunoinformatics, and reverse vaccinology have emerged as fundamental and indispensable approaches in the field of vaccine development (13). In the aforementioned analysis, the researcher can collect and analyze sequential data to build vaccines against various dangerous bacterial species (14).

The utilization of computer-aided techniques in vaccine development shows promise in establishing a theoretical framework for the creation of multi-epitope vaccine constructs that may effectively target various pathogenic species. This strategy involves identifying potential vaccine candidates within the entire proteome (15). Multi-epitope-based vaccinations mostly comprise several antigenic epitopes rather than solitary epitopes, so eliciting a robust and secure immune response within the host organism (16). Typically, the identification of anticipated epitopes relies on experimental data obtained on a case-by-case basis. The hepatitis C virus served as a prime illustration in the pursuit of developing a very effective recombinant vaccine. The development of epitope-based vaccine design has been ongoing for the past thirty years. In comparison to entire pathogen vaccinations, multi-epitope vaccines have lower inherent immunogenicity. However, the construction of epitope-based vaccines offers a safe means of inducing immunogenicity and effectively protecting the host (17).

The objective of the present study was to develop a multi-epitope vaccination construct targeting specific viral infections across their whole proteome. Furthermore, the study attempted to evaluate the docking contact between the suggested vaccine construct and various receptors on immune cells. Furthermore, molecular dynamic modelling was conducted to analyze the motion of the docked complexes, since this is of utmost importance for antigenic presentation, processing, and the induction of an appropriate immune response in the host against specific pathogens. The discovery has the potential to mitigate the challenges encountered in traditional vaccinology through the utilization of pan-genome-based vaccinology methods. This strategy can yield substantial cost and time savings in the vaccine formulation process.

## Research methodology

**Figure 1:**
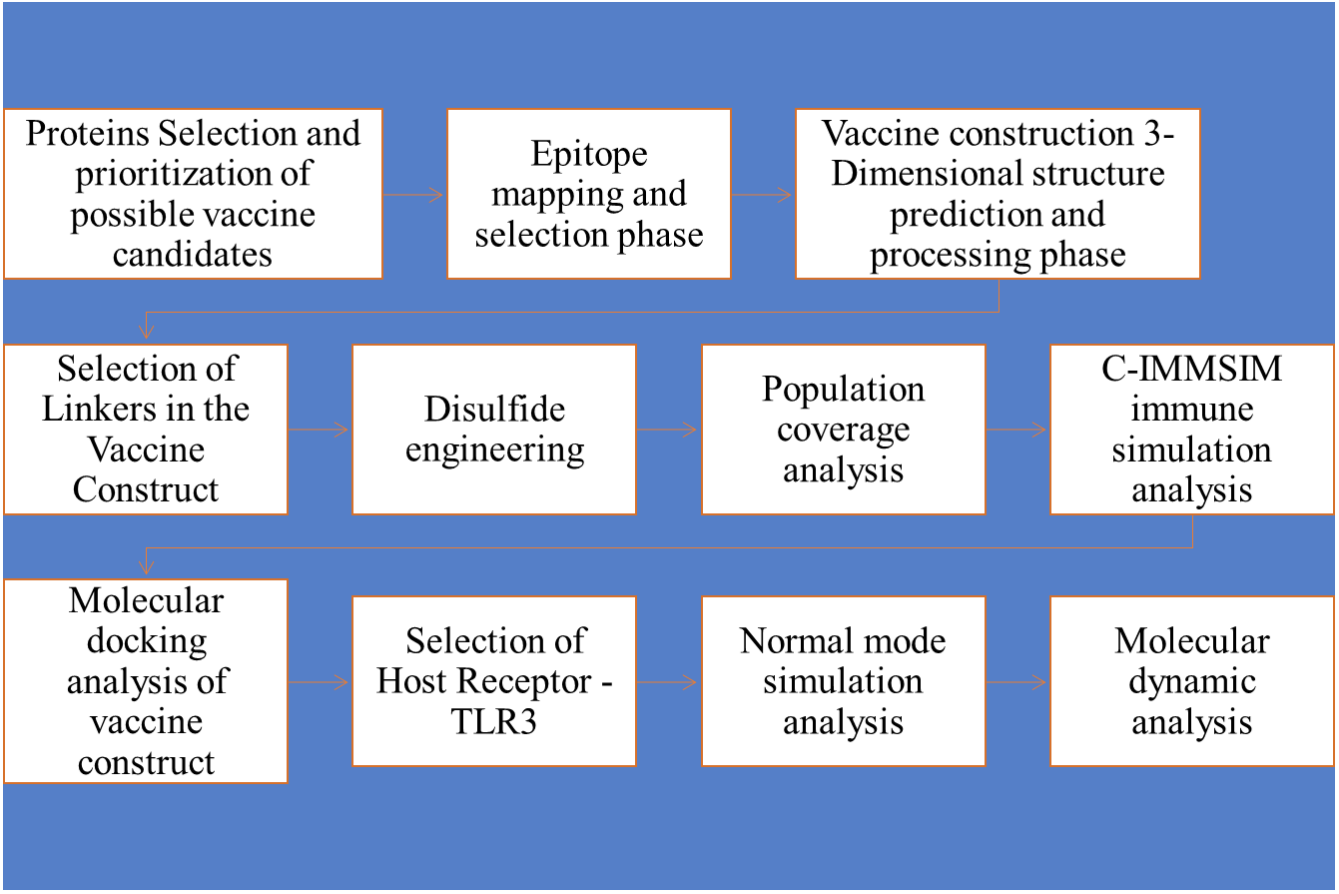
Methodology flow diagram, followed by the designing of a multi-epitopes vaccine candidate against WNV by applying several Immunoinformatics and biophysical approaches.

**Figure 1** Schematic representation of the study conducted for the multi-epitope vaccine construct.

### Proteins selection and prioritization of possible vaccine candidates (epitopes)

The investigation was initiated by conducting a comprehensive extraction of the proteome of WNV from the NCBI database, which was subsequently followed by the prioritization of potential vaccine targets (18). During the phase of vaccine target prioritization, the examination of physiochemical properties was conducted. This analysis involved examining many factors such as molecular weight, theoretical isoelectric point (pI), instability index, aliphatic index, and GRAVY. The ProtParam web server (19) was utilized for this purpose. A variety of computational tools were used to evaluate antigenicity, allergenicity, and toxicity using the following computational tools: VaxiJen 2.0 (20), Allerton (21) and ToxinPred (22). To identify potential vaccine candidates that are likely to exhibit antigenicity, lack allergenicity, and possess non-toxic properties, a rigorous selection process is necessary to mitigate the risk of triggering autoimmune responses. A BLASTp analysis was conducted to identify and eradicate duplicate proteins to reduce the risk of triggering auto-immune responses (23).

### Epitope mapping and selection phase

The multi-epitope-based vaccine construct is composed of a sequence of short peptides that overlap with each other. This design allows for an effective enhancement of the immune response due to the antigenic and immunogenic properties of the epitopes. The study utilized the NP_041724.2 polyprotein and YP_009164950.1 truncated flavivirus polyprotein NS1 prime proteins as antigenic and physio-chemically stable proteins to predict and map epitopes. B cell epitopes were predicted and selected using the “IEDB” online web server (24). In addition, the epitopes of B-cells were employed for the prediction of T-cell epitopes in the major histocompatibility complex (MHC-I and MHC-II). The predicted epitopes were ranked based on the lowest percentile rank (15). The epitopes that were predicted were further evaluated for their antigenicity, allergenicity, water solubility, and toxicity using VaxiJen 2.0, AllerTop 2.0, and the Solubility Peptide Calculator available at https://pepcalc.com/peptide-solubility-calculator.php. Afterwards, we proceeded to choose epitopes that were deemed to be potentially antigenic, non-allergic, non-toxic and had good water solubility (25).

### Vaccine construction 3-dimensional structure prediction and processing phase

The utilization of multi-epitope-based vaccines represents a highly promising strategy within the field of reverse vaccinology (26). The field of vaccinology research (RV) encompasses contemporary approaches that aim to identify potential vaccine candidates within the core proteome of pathogens, applying various bioinformatics and immunoinformatics technologies. The multi-epitope vaccine design primarily consists of diverse antigenic epitopes that are non-allergenic, highly soluble in water, and non-toxic (27). During the construction phase of the multi-epitope-based vaccine (MEVC), the shortlisted epitopes were connected using GPGPG linkers. Furthermore, to improve the effectiveness of the vaccine construct, the designed vaccine construct was linked with the cholera toxin-B subunit adjuvant, which is known to induce immune efficacy (28).

### Choice of linker in the vaccine construct

The utilization of GPGPG linkers in our vaccine design is a deliberate choice, guided by their well-documented efficacy in maintaining structural stability and epitope presentation. GPGPG linkers are short, flexible sequences that provide sufficient space between epitopes, thereby reducing structural hindrance and potential steric clashes. This spatial separation is crucial for the independent folding and processing of each epitope, ensuring that each component of the vaccine is effectively recognized by the immune system (29). The flexibility offered by GPGPG linkers also facilitates the physical separation of functional domains within the vaccine construct, minimizing interference in their respective immune elicitation roles. This is particularly important in multi-epitope vaccines, where the goal is to present multiple antigens to the immune system in a manner that mimics natural infection closely (16). Alternative linkers, such as EAAAK could theoretically be used in vaccine constructs and may influence the vaccine’s efficacy differently. We used an EAAAK linker to join an adjuvant with a vaccine construct The specifically used linkers have many advantages for example, they are known for their rigid helical structure, which could enhance the structural stability of the construct but might limit the flexibility and independent orientation of linked epitopes. On the other hand, EKK linkers, owing to their positive charge, could potentially improve the solubility of the vaccine construct but may also affect the overall immunogenicity due to their shorter length and less pronounced structural separation capabilities (30). Ultimately, the choice of GPGPG was predicated on achieving an optimal balance between structural flexibility, solubility, and the effective presentation of epitopes to the immune system. This careful consideration ensures that our vaccine construct can elicit a broad and robust immune response, a critical factor in the successful development of a vaccine against West Nile Virus (31).

Following the development of the multi-epitope vaccine construct, the vaccine construct underwent subsequent processing for the examination of its physiochemical properties. This analysis was conducted using the ProtParam tool (19), which can be accessed at https://web.expasy.org/protparam/. In addition, the vaccine design underwent examination for antigenicity, allergenicity, toxicity, and water solubility utilizing VaxiJen 2.0, AllerTop 2.0, ToxinPred, ProtParam, and INNOVAGEN web tools (32). The three-dimensional structure of the vaccine construct was created utilizing the “Scratch protein predictor online tool” (28). The vaccine structure underwent additional refinement using the GalaxyRefine web-based tool, the refined structure of the vaccine construct was then selected for further processing (33).

### Disulfide engineering

Following the refining of disulfide linkages, engineering was conducted using the web-based program Design 2.0. This resulted in the creation of a mutant structure, denoted as MEVC, whereby all enzymatic sensitive residues were substituted with cysteine amino acid residues (34).

### Population coverage analysis

The examination of population coverage was conducted using the Immune Epitope Database (IEDB) online database (24). This database was utilized to analyze the selected epitopes for the vaccine construct in terms of their coverage across global populations and individual countries (35).

### C-IMMSIM immune simulation analysis

The analysis of immune simulation was conducted using in silico methods, specifically through the utilization of the C-IMMSIM internet web server (36). In the context of C-IMMSIM immunological simulation analysis, distinct immune responses of B and T-cells were found towards the vaccine design (37).

### Molecular docking analysis of vaccine construct

Molecular docking analysis is a computational-based analysis that is mainly used for binding interaction analysis between ligand and receptor molecules (Vaccine and immune cells receptors (38, 39), In the molecular docking analysis phase immune cell receptor molecule, was selected as a receptor for the design of vaccine construct, and pdb structure of the Toll-like receptor-3 (TLR-3) was retrieved from protein data bank (pdb) (40) and prepare for docking with vaccine using UCSF chimaera tool (41), for molecular docking analysis cluspro 2.0 web tool was utilized (38), the target receptor TLR-3 plays a vital role in the immunogenicity in the pathogenesis of viral pathogenic species (42). Both the vaccine and receptor PDB files were uploaded to the cluspro 2.0 web server and submitted for docking analysis. The “cluspro 2.0 webserver” generated top-10 docked complexes of vaccine molecule with target receptor (TLR-3).

### Selection of host receptor - TLR3

In the design of our proposed multi-epitope vaccine against West Nile Virus (WNV), we specifically targeted Toll-like receptor 3 (TLR3) due to its critical role in the innate immune system’s response to viral infections. TLR3, a pattern recognition receptor, identifies double-stranded RNA (dsRNA), a molecular pattern associated with viral replication, including that of flaviviruses such as WNV. The activation of TLR3 initiates a cascade of antiviral defenses, notably the induction of type I interferon responses, which are paramount in controlling viral load and spread early in the infection. Moreover, TLR3’s involvement in the maturation and activation of dendritic cells underscores its importance in bridging innate and adaptive immunity, thereby enhancing the antigen presentation process and the subsequent activation of a targeted immune response (43).

### Normal mode simulation analysis

To explore the dynamic motion of the docked complex normal mode simulation analysis was done using the iMOD normal mode analysis server. In the mode simulation analysis, the stability and three-dimensional structure of the construct with the docked structure were analyzed. For the normal mode simulation analysis the pdb file of the docked complex was used having the lowest binding energy score (15, 44).

### Molecular dynamic analysis

Molecular dynamic simulation analysis was applied for the dynamic behavior analysis of docked molecules in the specific period of 100 nanoseconds (ns). The MD simulation analysis was done by using AMBER 21 packages (45). For simulation analysis top-1 docked compound was selected which was generated by cluspro 2.0, the selection was based on the lowest binding energy score. The simulation was done mainly in three phases, system preparation, pre-processing, and trajectory analysis phase. In the trajectories analysis phase mainly three trajectories “root mean square deviation (RMSD)”, “root mean square fluctuation (RMSF)” and radius of gyration (RoG) were created. In root mean square deviation analysis time-dependent deviation was analyzed while in root mean square fluctuation residues dependent fluctuation of the residues of the protein was analyzed. Moreover, the docked complex compactness and relaxation were assessed through the radius of gyration analysis as the compactness is crucial for docked complexes for appropriate stability (46).

## Results

### Subtractive proteomic analysis

In subtractive proteomic analysis, we extracted two proteins of WNV using accession numbers>NP_041724.2 and >YP_009164950. The proteins were subjected to physiochemical properties analysis and both the proteins were predicted with stable physiochemical properties as tabulated in **Table 1**.

**Table 1:** Physiochemical properties analysis of the selected proteins.

	Proteins accession number	Physiochemical properties Analysis						
		Number of amino acids	Molecular weight (kDa)	Theoretical pI		Instability index	Aliphatic index	GRAVY
1	>NP_041724.2	3430	38.010982	8.63		34.02	86.14	-0.15
2	>YP_009164950	1191	13.02591	8.66		36.55	80.4	-0.162

The physiochemical stable proteins were considered for further subtractive proteomics analysis, in antigenicity analysis both proteins were predicted as probable antigenic with 0.5407 and 0.6015, >NP_041724.2, >YP_009164950 respectively, in water solubility and allergenicity analysis both the proteins were predicted good water soluble and non-allergic. In homology analysis, the selected proteins were predicted as non-similar to human and human microbiota analysis. Finally, the selected epitopes were selected for the epitopes prediction and prioritization phase.

### Epitope prediction and prioritization analysis phase

In the epitopes prediction and prioritization phase B cell epitopes were predicted from the selected proteins in this phase both B and T cells (MHC-I and MHC-II) epitopes were predicted the predicted epitopes are mentioned in the following, **Table 2** mentions B and B cells derived T-cells Epitopes. The B cells peptide was then used for the T-cells (MHC-I and MHC-II) Prediction, based on the lowest percentile ranks the epitopes were prioritized and selected for further analysis as described in the following **Table 3**.

**Table 2:** B-cell epitopes, predicted from shortlisted proteins.

S.NO	Selected Proteins	B-cells Peptides
1	>NP_041724.2 polyprotein	NRRSTKQKKRGGTA
		TRAACPTMGEAHNEKRAD
		PTTVESHGKIGATQA
		IDGPETEECPTANRAW
		AGPRSNHNRRPGYKTQNQGPWDEGRV
		SCEHRGPAARTTTESGK
		DITWESDAEITGSSER
		GKNVKNVQTKPGVFKTPEGEIGA
		LDYPTGTSGSPIVDKNG
		ATPPGTSDPFPESNAPISDMQTEIPDRAWNT
		KSYETEYPKCKNDD
		SAITAASAAQRRGRIGRNPSQVGDEYCYGGHTNEDDSNFA
		FDGPRTNTILEDNNE
		GWQAEAMRSAQRRT
		ITEVDRSAAKHARREGNITGGHPVSRGT
		RVQEVKGYTKGGPGHEEPQLV
		KKTWKGPQFEEDVNLGSGTRAV
		SSTWHQDANHPYRTWNYH
		KVDTKAPEPPEGV
		SAITAASAAQRRGRIGRNPSQVGDEYCYGGHTNEDDSNF
		ITEVDRSAAKHARREGNITGGHPVSRGT
		RVQEVKGYTKGGPGHEEPQLV
		KKTWKGPQFEEDVNLGSGTRAV
		SSTWHQDANHPYRTWNYH
		QNQWKNAREAVEDPK
2	>YP_009164950.1 truncated flavivirus	NRRSTKQKKRGGTA
		PTTVESHGKIGATQA
		EVTVDCEPRSGIDT
		AGPRSNHNRRPGYKTQNQGPWDEGRV
		SCEHRGPAARTTTESGK
		RVLGHPGGPSQEVDGQDQHSSYHACTPSPSVW
		NRRSTKQKKRGGTA

**Table 3:** MHC (I & II) predicted epitopes from B-cell epitopes with the lowest percentile rank.

MHC-I	Lowest percentile rank	MHC-II	Lowest percentile rank
NRRSTKQKK	2	NRRSTKQKKRGGT	49
TMGEAHNEK	0.67	RAACPTMGEAHNEK	25
HGKIGATQA	2.1	VESHGKIGATQA	3.4
ETEECPTANR	0.09	IDGPETEECPTANRA	54
NQGPWDEGR	3	YKTQNQGPWDEGRV	40
SCEHRGPAAR	3.3	SCEHRGPAARTTT	9.6
ESDAEITGSS	3.7	WESDAEITGSSE	2.4
KNVKNVQTK	0.48	GKNVKNVQTKPGV	13
GTSGSPIVDK	0.05	PTGTSGSPIVDK	11
ESNAPISDM	0.24	FPESNAPISDMQTE	13
KSYETEYPK	0.02	KSYETEYPKCKN	8.9
TAASAAQRR	0.05	SAITAASAAQRRG	1.8
DGPRTNTIL	1.2	FDGPRTNTILEDN	8.5
EAMRSAQRR	0.07	GWQAEAMRSAQRR	18
EAMRSAQRR	0.06	EGNITGGHPVSR	3.3
RVQEVKGYTK	0.07	RVQEVKGYTKGG	7.4
GPQFEEDVNL	0.57	GPQFEEDVNLGSG	3.9
TWHQDANHPY	0.83	SSTWHQDANHPY	0.81
KAPEPPEGV	0.32	VDTKAPEPPEGV	42
TAASAAQRR	0.05	SAITAASAAQRRGR	2.1
NITGGHPVSR	0.25	EGNITGGHPVSR	3.3
RVQEVKGYTK	0.07	RVQEVKGYTKGGP	20
GPQFEEDVNL	0.57	GPQFEEDVNLGS	3.3
TWHQDANHPY	0.83	SSTWHQDANHPY	0.81
NQWKNAREA	1.3	QNQWKNAREAVEDPK	16
NRRSTKQKK	2.4	NRRSTKQKKRGG	24
HGKIGATQA	2.1	TVESHGKIGATQA	4.2
EVTVDCEPR	0.06	EVTVDCEPRSGI	9.5
RPGYKTQNQ	1.9	RPGYKTQNQGPWD	34
SCEHRGPAAR	3.3	SCEHRGPAARTTT	9.6
HACTPSPSVW	0.03	HSSYHACTPSPSVW	15
NRRSTKQKK	2	NRRSTKQKKRGGT	49
TMGEAHNEK	0.67	RAACPTMGEAHNEK	25
HGKIGATQA	2.1	VESHGKIGATQA	3.4
EVTVDCEPR	0.06	IDGPETEECPTANRA	54
RPGYKTQNQ	1.9	YKTQNQGPWDEGRV	40
SCEHRGPAAR	3.3	SCEHRGPAARTTT	9.6
HACTPSPSVW	0.03	WESDAEITGSSE	2.4

### Epitopes screening phase

The predicted epitopes underwent further screening, including MHC prediction, antigenicity, allergenicity, toxicity, and water solubility analysis. Only the probable antigenic, well-binding to DRB*0101, water-soluble, and non-toxic epitopes were chosen for the design of the epitope-based vaccine construct. The shortlisted epitopes are presented in **Table 4**.

**Table 4:** Characteristics of selected epitopes for designing of epitopes-based vaccine construct.

Shortlisted epitopes	Calculated DRB*0101 Allele binding score	Antigenicity	Allergenicity	Toxicity	Water solubility
NRRSTKQKK	30.06	0.2751	Non-allergens peptides	Non-toxic peptides	Good water-soluble peptides
KSYETEYPK	37	0.0578			
TAASAAQRR	22	1.1437			
NRRSTKQKK	30	0.2751			

### Epitopes vaccine construction MEVC and evaluation

In the epitope-based vaccine construction phase, the final shortlisted epitopes were utilized to create a multi-epitope vaccine construct. This construct primarily comprised four selected epitopes with varying lengths, connected by GPGPG and EAAAK linkers, along with an adjuvant. The adjuvant was linked via the EAAA linker. Following the assembly of the linear epitope-based vaccine construct, it underwent evaluation for physicochemical properties analysis.

**Figure 2:**
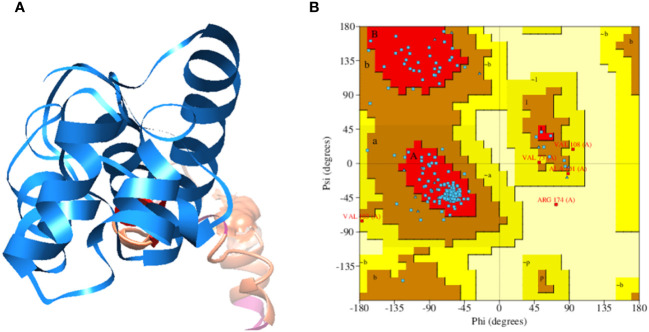
**(A)** Multi-epitopes based vaccine construct against West Nile Virus, consisting upon selected epitopes, GPGPG and EAAAK linkers and Cholera Toxin B subunit adjuvant. **(B)** Ramachandran Plot of the tertiary structure of vaccine construct.

The analysis indicated that the designed vaccine construct consists of 180 amino acid sequences, with a molecular weight of 19.81774 kilodaltons (KDa), a Theoretical pI of 9.91, and an Aliphatic index of 66.28. The instability evaluation yielded a value of 40.78, suggesting that the vaccine construct is classified as stable. Subsequently, the designed sequence of the vaccine construct was utilized for three-dimensional structure prediction. **Figure 2A** illustrates a 3D representation of the multi-epitope vaccine construct (MEVC). Furthermore the tertiary of the vaccine construct was validated through Ramachandran analysis, in the Ramachandran Plot statistics we observed that 128 (83.1%) amino acid residues are present in the most favored regions, while 21 (13.6%) amino acid residues are predicted in the additional allowed regions, moreover, 4 (2.6%) number of amino acids residues were observed in the Generously allowed regions and 1 (0.6%) several amino acids residues were observed in the Disallowed regions. Next 154 (100.0%) Non-glycine and non-proline residues were also observed. Further 14, 10 glycine and proline residues respectively observed and 2 End-residues (excl. Gly and Pro), the above statistic was based on a total number of 180 amino acid residues of the vaccine construct and it represents that the vaccine construct structure is stable, as the Ramachandran analysis graph is mentioned in **Figure 2B**.

**Figure 3:**
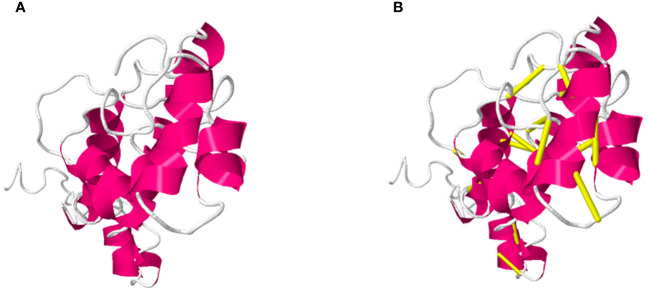
Demonstrate the Original **(A)** and mutated structure of the designed vaccine construct, in **(B)** the yellow color sticks in the structure represent mutated residues in the mutant structure.

Furthermore, in disulfide engineering the original structure is mutated and replaced enzymatic sensitive amino acid residues with cysteine amino acid (Leu 4-Asn 35, His 20- Asn 25, Ala 17-Lue 29, Cys 30- Asn 35, Gln 37-Lue 41, Arg50-Gly 75, Tyr 97-Cys 150, c\Cys 107- Ala 127, Pro 151-Thr 157) pairs of residues were selected to be mutated. The original and mutated structures of the designed vaccine construct are presented in following **Figures 3A, B**.

**Figure 4:**
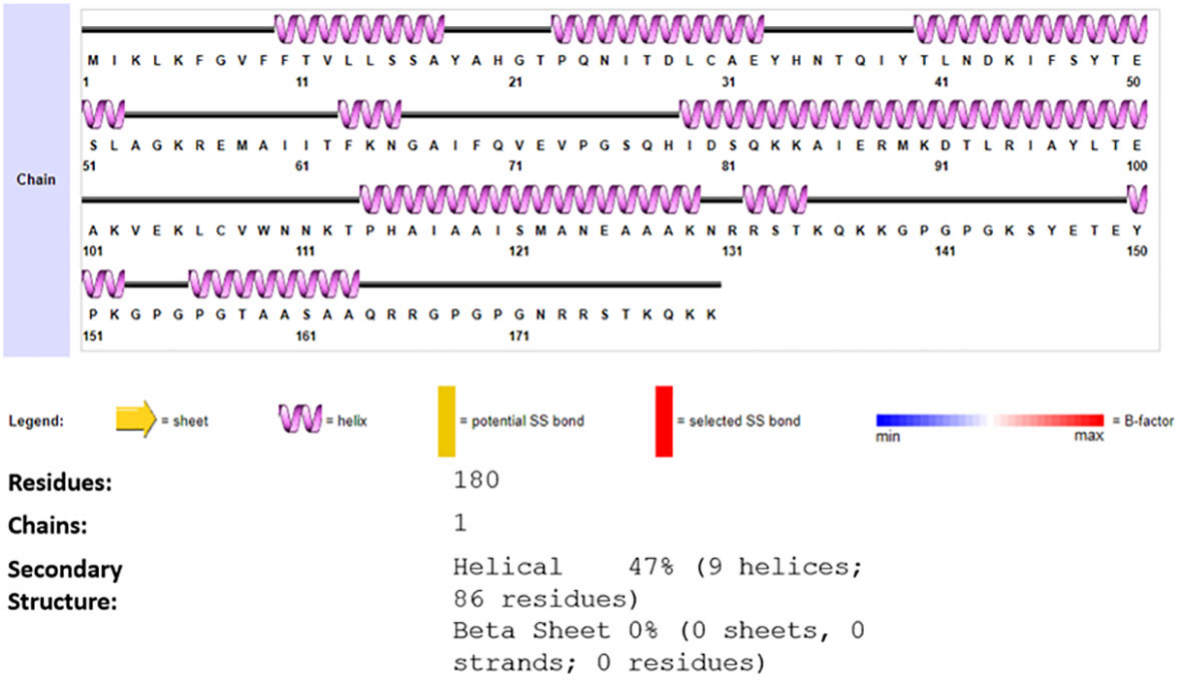
Predicted secondary of vaccine construct, the different colors Legend demonstrate sheet alpha helix etc. of the generated structure of vaccine construct.

Next Secondary structure was generated, and the secondary structure predicted 180 residues, 1 chain 9 (47%) helices and 0 beta sheets, **Figure 4** presented the description of the secondary structure of the vaccine construct.

### Cloning of MEVC and codon optimization

The sequences were first converted to DNA sequences.

**Figure 5:**
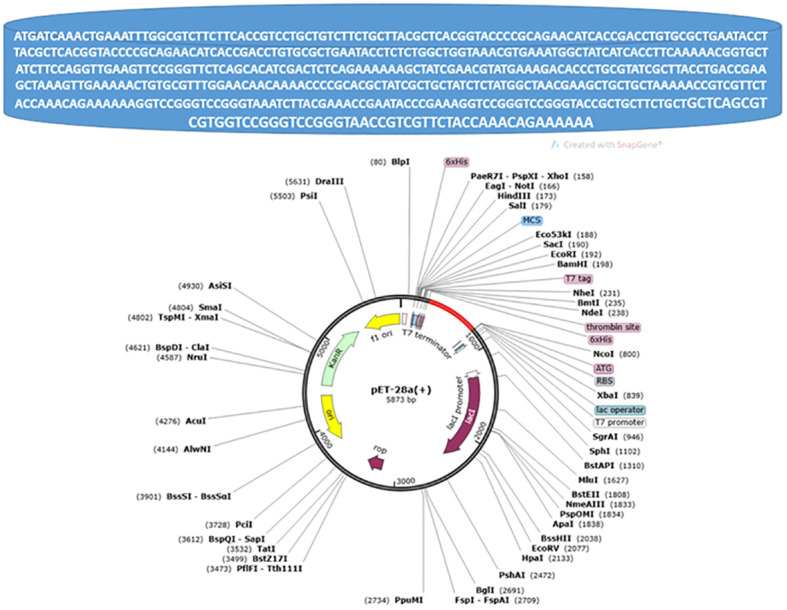
Pet28a vector map, the red color in the circle represents the inserted improved DNA sequence.

“ATGATCAAACTGAAATTTGGCGTCTTCTTCACCGTCCTGCTGTCTTCTGCTTACGCTCACGGTACCCCGCAGAACATCACCGACCTGTGCGCTGAATACCTTACGCTCACGGTACCCCGCAGAACATCACCGACCTGTGCGCTGAATACCTCTCTGGCTGGTAAACGTGAAATGGCTATCATCACCTTCAAAAACGGTGCTATCTTCCAGGTTGAAGTTCCGGGTTCTCAGCACATCGACTCTCAGAAAAAAGCTATCGAACGTATGAAAGACACCCTGCGTATCGCTTACCTGACCGAAGCTAAAGTTGAAAAACTGTGCGTTTGGAACAACAAAACCCCGCACGCTATCGCTGCTATCTCTATGGCTAACGAAGCTGCTGCTAAAAACCGTCGTTCTACCAAACAGAAAAAAGGTCCGGGTCCGGGTAAATCTTACGAAACCGAATACCCGAAAGGTCCGGGTCCGGGTACCGCTGCTTCTGCTGCTCAGCGTCGTGGTCCGGGTCCGGGTAACCGTCGTTCTACCAAACAGAAAAAA” with (0.9607476511968707) codon adaption value “CAI-Value of the improved sequence” and (49.44) GC-Content of the improved sequence, The improved DNA sequence then inserted in pET-28a vector as pet28a vector map is presented in the following **Figure 5**.

### Interaction analysis

**Figure 6:**
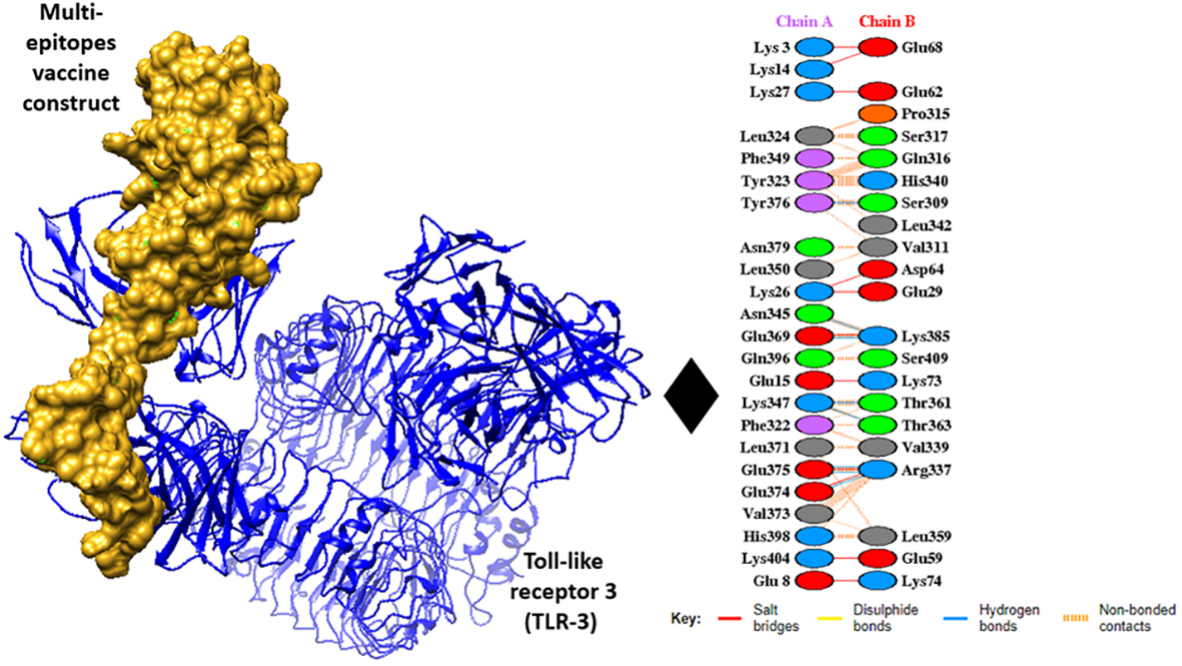
Intermolecular docked confirmation of vaccine with Toll-like receptor 3 (TLR3), and interactive amino acid residues.

For interaction analysis and the binding ability of MEVC to immune cell receptors molecular docking was performed, the docking analysis was performed through the online cluspro 2.0 web tool, and the tool generated the Top 10 docked complexes. **Table 5** mentions docked complexes with the lowest binding energy score. The Top 1 docked complex was then subjected to molecular dynamic simulation analysis. The intermolecular docked confirmation is represented in **Figure 6**.

**Table 5:** Top- 10 generated docked complexes of vaccine with TLR-3).

Cluster	Members	Representative	Weighted Score
**0**	91	Center	-924.4
		Lowest Energy	-938.2
**1**	89	Center	-791.5
		Lowest Energy	-960.3
**2**	60	Center	-915.0
		Lowest Energy	-952.1
**3**	49	Center	-878.4
		Lowest Energy	-878.4
**4**	45	Center	-983.5
		Lowest Energy	-983.5
**5**	42	Center	-859.1
		Lowest Energy	-859.1
**6**	35	Center	-750.9
		Lowest Energy	-910.5
**7**	28	Center	-827.8
		Lowest Energy	-891.4
**8**	27	Center	-789.4
		Lowest Energy	-839.9
**9**	23	Center	-846.7
		Lowest Energy	-929.3
**10**	20	Center	-789.2
		Lowest Energy	-834.6

### Normal mode molecular dynamic simulation

**Figure 7:**
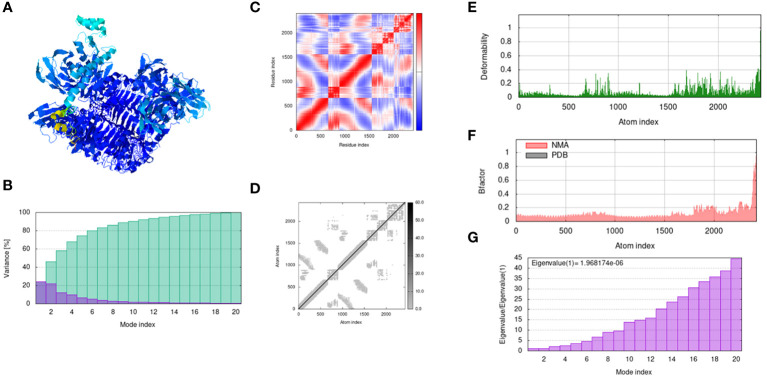
Output trajectories of normal mode simulation analysis of the vaccine and TLR-3. **(A)** complex; **(B)** Variance; **(C)** covariance index; **(D)** elastic network analysis; **(E)** deformability; **(F)** B-factor; **(G)** eigenvalue.

The IModS webserver does the precarious analysis of the docked structure by adjusting the force field of the docked complex concerning different interval times. The findings represent less deformation at each residue level. The Eigenvalues value of the complex is 1.96817e − 06. Next, the Lowest RMSD and highest co-related regions in the heat maps predicted that there are better interactions of each residue. The following **Figure 7** explains the outcome trajectories of the normal mode simulation analysis. In the following trajectories, the structure assessment of the docked complexes was examined in **Figure 7A** the mobility and structure strength of the docked complexes have been analyzed, and predicted that there is proper binding strength between the vaccine and receptors. In the structural variances analysis of the docked complexes, we observed that the structures of the docked complexes are compact and no abrupt variances occur during simulation time the variances plot is mentioned in **Figure 7B** while the covariance plot is presented in **Figure 7C**. The elastic network related to the atom is mentioned in **Figure 7D**. Furthermore deformability of the docked structure was also analyzed and we observed that there is no deformation occurring in the docked complexes, the deformability plot is mentioned in **Figure 7E**. The beta factor plot of the NMA is presented in **Figure 7F** and the graph presents that the docked complexes are stable and compact. Moreover, the eigenvalues plot is presented in Figure.

### Molecular dynamic simulation analysis

**Figure 8:**
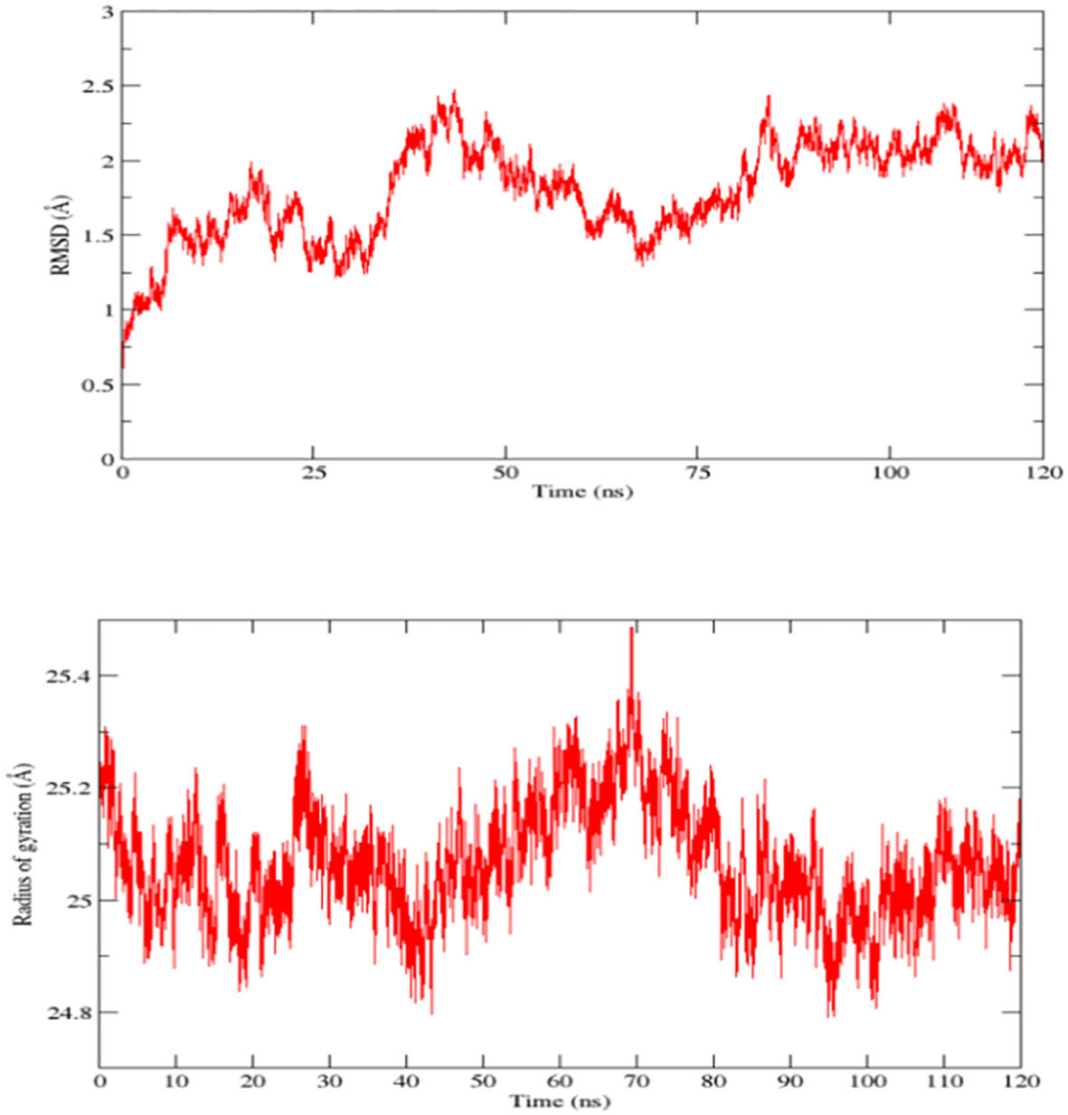
Root mean square deviation (RMSD) and radius of gyration (RoG) plots of docked complexes (Vaccine and Toll-like receptors 3), top one represents RMSD while the bottom one represents RoG.

In molecular dynamic simulation analysis, the dynamic movement of the docked complexes was analyzed, and the trajectories of MD simulation were analyzed on the base of kinetics and total net binding energy (RMSD and radius of gyration). In MD simulation RMSD was analyzed as RMSD is the measurement of the average distance between the backbone atoms of the protein. RMSD plots predicted the highest deviation at 80 ns but at the end time of the simulation, the plot became stable and showed stability as the RSMD graph is presented in **Figure 8** top plot. Next radius of gyration analysis was performed for competence and relaxation of the docked complexes at 70 nanoseconds the RoG plot showed fluctuation however at the end of simulation time we observed stability in the RoG plot which predicted that the vaccine and immune receptor are compact as the RoG graph is presented in **Figure 8** bottom plot.

### C-immune simulation analysis

**Figure 9:**
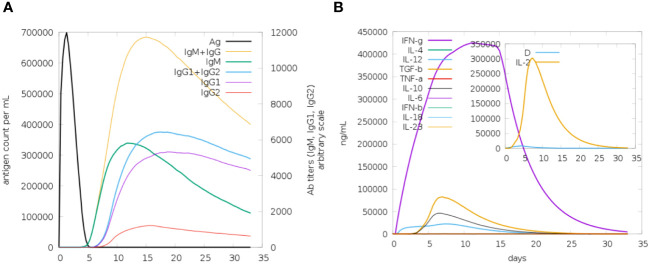
**(A)** The immunoglobulins and the immunocomplexes **(B)** Different concentration of cytokines and interleukins. Inset plot Shows danger signal together with leukocyte growth factor interleukin (IL-2).

In the c-immune simulation analysis, we observed the immune-inducing ability of the vaccine construct, the server predicted that the MEVC could produce a strong immune response in the host body against the designed vaccine construct. The immune system response was detected in the form of humoral and cellular immunity **Figure 9A** represents antibody response toward the designed vaccine in the form of IgM and IgG followed by IgG1 and IgG2 as overall the immune response is presented by different colors peaks. Next cellular immune was also observed in the form of interferon and several other types of cytokines and tumor growth factor beta and tumor necrosis factor a (TNFa) as the cellular immune response as presented in **Figure 9B**.

### Population coverage analysis

**Figure 10:**
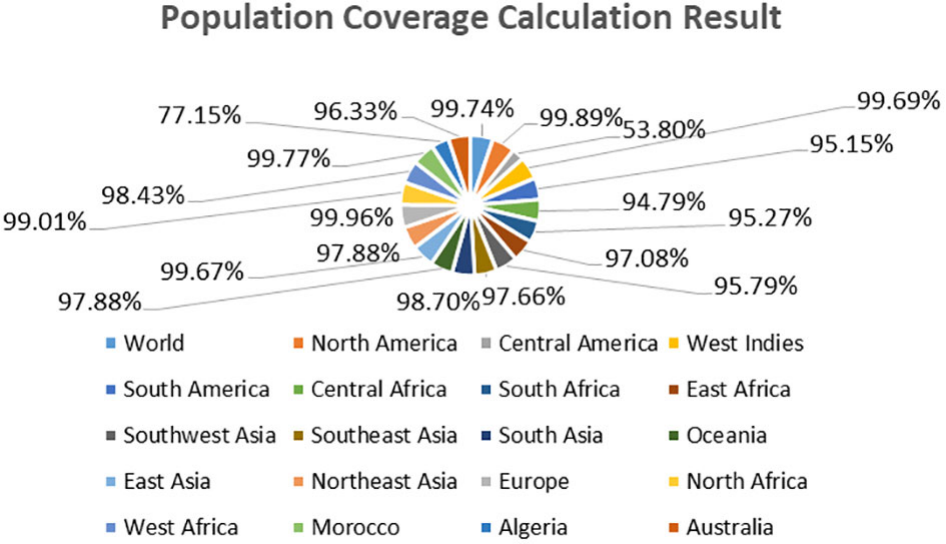
Population coverage results of selected epitopes for vaccine construction.

In the population coverage analysis, we observed that the selected epitopes can cover the worldwide population (Class combined “MHC-I and MHC-II) as we predicted that World, North America, Central America, West Indies, South America, Central Africa, South Africa, East Africa, Southwest Asia, Southeast Asia, South Asia, Oceania, 99.74%, 99.89%,53.80%, 99.69%, 95.15%, 94.79%, 95.27%, 97.08%,95.79%., 97.66%, 98.70%, 97.88% respectively while the other countries population coverage are presented in **Figure 10**. However different other countries and their population coverage are tabulated in **Supplementary Table 1 (S1)**.

## Discussion

A multitude of WNV cases have been reported throughout the United States, predominantly observed during the summer season. It is noteworthy that the annual incidence of WNV exhibits global variability, primarily attributed to periodic outbreaks (47). The challenge of addressing WNV lies in the lack of a suitable commercial vaccine, as the availability of a vaccine would provide a valuable and secure means of eradicating infectious diseases on a global scale. The process of vaccine development necessitates a substantial allocation of labor and financial resources (38). The application of reverse vaccinology, bioinformatics, and immunoinformatics has significantly alleviated this burden. Presently, researchers employ reverse vaccinology pipelines to discern numerous antigenic vaccine targets against various bacterial and viral pathogens by analyzing the proteins of said pathogens (48). Reverse vaccinology mainly uses the expressed genomic sequences of the pathogens for the identification of vaccine candidates, as in pasture base vaccine development vaccines are created by using pathogenic organisms which can cause harmful effects during vaccine formulation, but in the case of reverse vaccinology the experimental scientist the purified and possible vaccine candidate for vaccine formulation (49).

The absence of a vaccine targeting the aforementioned virus can be attributed to the relatively low rates of mortality and morbidity associated with it. However, it is imperative to acknowledge that our global community remains persistently vulnerable to numerous potential epidemics and endemics. Notable instances include the emergence of Bolivian Hemorrhagic fever and the coronavirus infection in 2019 (50). The investigation does not necessitate exclusive attention on the outer membrane complete proteins of the pathogens. It is worth noting that through the utilization of the immunoinformatics approach, researchers have successfully identified potential multi-antigenic vaccine targets that exhibit a high degree of antigenicity. These findings hold significant promise for the advancement of vaccine development (51). The potential vaccine should be probable antigenic, physiochemical stable, good water soluble, non-allergic, nontoxic and good water-soluble. The purpose of the said characteristics is to target only probable antigenic epitopes, physiochemical stable, good water soluble non-allergic and nontoxic respectively, the only antigenic epitopes can elicit an immune response so only antigenic epitopes were considered for further filtering, also to avoid allergic and toxic reaction toward vaccine candidates. the allergic and toxic epitopes were removed (28). In epitopes prediction specifically two antigenic proteins >NP_041724.2 polyprotein and >YP_009164950.1 truncated flavivirus were used for epitope prediction using the IEDB online web server, and the epitopes were selected for further filters based on the lowest percentile score (24).

The viral poly proteins mainly play a vital role in the budding of the virus by binding to the host cell membrane and gathering the viral genomic material (DNA or RNA) into the nucleocapsid that produces the core of a virion particle. The WNV polyprotein is the first virus protein that is produced by the infected cells, but the main role of the target protein plays an important role in the virus assembly formation, and it can also block the process of host cell apoptosis during the viral replication process. Next, the second target protein of the WNV mainly plays a vital role in the process of WNV propagation, with attachment, entrance, replication, assembly formation and release of complete viral particles. So we target these proteins specifically to predict the epitopes and reduce the viral propagation by generation of immune responses (52).

The selected epitopes were further filtered through various subtractive immunoinformatics filters, like antigenicity, allergenicity, water solubility and toxicity, to activate proper immune responses and avoid allergic and toxic reactions toward the vaccine candidate. The probable antigenic epitopes could evoke proper immune reaction inside the host against specific pathogens, next allergic response with the vaccine is one of the main concerns, to avoid the allergic response, all the allergic epitopes were discarded using AllerTOP v. 2.0 online web server and only the non-allergic epitopes were processed further. Followed by allergenicity prevention from the toxic immune response is also vital to prevent the host from a toxic reaction. Utilizing all the immunoinformatics approaches probable antigenic and good vaccine target epitopes were selected for a multi-epitope vaccine designed against target pathogens (23, 25).

Multi-epitope vaccine construct is mainly composed of multi-antigenic peptides rather than whole proteins and can be considered a promising strategy against tumors and viral infections. The epitopes-based vaccine was designed by joining the selected epitopes through GPGPG linkers and joined with the Cholera Toxin B subunit adjuvant. The GPGPG linkers were used to avoid flexibility in the vaccine construct while the adjuvant was used for inducing immunodeficiency of the vaccine to construct. After designing the sequences of the vaccine construct, and being subjected to Scratch Protein Predictor for structure prediction the vaccine construct, the 3D structure was then refined through another software GalaxyRefine and all the present loops from the structure to the structural stability and competence. Next, the structure minimization of the vaccine was done before docking analysis to optimize the active sites of the target receptors (53).

The crucial aspect in the induction of an appropriate immune response against pathogens lies in the intricate interplay between vaccine construction and immune cell receptors. The interaction analysis involved conducting a Molecular docking analysis to examine the vaccine’s binding affinity with the TLR-3 molecule. The docking analysis was conducted utilizing the ClusPro 2.0 web tool and predicted the interaction of vaccine construct with the target immune cell receptor, ClusPro 2.0 is a widely recognized webserver for molecular docking analysis. The tool was employed to generate a set of Top-10 models based on their respective binding energy scores, thereby providing valuable insights into the docking process. The assessment of the vaccine construct’s stability in conjunction with the receptor molecule is of utmost importance. To fulfil this objective, we conducted an analysis utilizing normal mode simulation and molecular dynamic simulation techniques were employed. Additionally, we examined the deformability of the structure throughout the simulation time and predicted that the vaccine construct and immune cell receptor have proper binding affinity in a dynamic environment. Afterwards, C-immune simulation analysis of the vaccine construct has the potential to elicit an immune response against WNV through the activation of both B and T-cells, thereby enhancing the immune system’s ability to combat the virus (54). Researchers can utilize the proposed vaccine construct during the development of experimental vaccines targeting specific pathogens. The inclusion of experimental validation will significantly contribute to the theoretical foundation of the vaccine.

## Conclusion

The organism that is responsible for the transmission of the WNV is primarily mosquitoes. This virus induces a range of clinical manifestations, such as elevated and fluctuating body temperature, Cephalalgia, cervical rigidity, altered mental state, profound confusion, unconsciousness, involuntary muscle contractions, convulsions, muscular debility, visual impairment, sensory numbness, and motor paralysis. These symptoms have been observed within the United States. To propose a potentially effective strategy for mitigating the presence of disease-causing surface-associated proteins, a theoretical foundation for a vaccine construct was devised utilizing an advanced bioinformatics methodology known as next-generation sequencing. The vaccine construct that was designed consisted of various epitopes, adjuvants, and linkers. An analysis of its physiochemical properties, structure, antigenicity, and allergenicity was conducted. The designed vaccine construct, which was deemed potentially antigenic, non-toxic, and non-allergic, was then utilized for further analysis. Utilizing biophysical methodologies, including docking and simulation analysis, we investigated the binding mode of the vaccine with TLR-3. Our analysis revealed promising results, suggesting that the vaccine construct possesses the capability to bind with the desired target receptors. Moreover, our analysis of the C-immune system revealed a significant induction of immune response against the targeted pathogens upon administration of the vaccine construct. Additionally, the designed vaccine demonstrated the ability to stimulate the production of various interferons. Nevertheless, it is highly advisable to conduct an experimental study to further substantiate the *in-silico* findings.

## Data availability statement

The original contributions presented in the study are included in the article/**Supplementary Material**. Further inquiries can be directed to the corresponding author.

## Author contributions

AK: Writing – original draft.
