# Supplementary material for: Immunoinformatics assisted profiling of West Nile virus proteome to determine immunodominant epitopes for the development of next-generation multi-peptide vaccine
Source: Front Immunol. 2024 May 10;15:1395870. doi: 10.3389/fimmu.2024.1395870 (PMC11116617; doi:10.3389/fimmu.2024.1395870)
Supplement: Supplementary file 1 [file Table_1.docx]

**Immunoinformatics Assisted Profiling of West Nile Virus Proteome to Determine Immunodominant Epitopes for the Development of Next-Generation Multi-Peptide Vaccine**

Alaa Karkashan

Department of Biology, College of Sciences, University of Jeddah, Jeddah 21959, Saudi Arabia; [askarkashan@uj.edu.sa](mailto:askarkashan@uj.edu.sa)

Supplementary Table 1 (S1). World and different countries wise population coverage analysis results predicted by IEDB Analysis Resource data base.

| **population/area** | **Class I** | | | **Class II** | | | **Class combined** | | |
| --- | --- | --- | --- | --- | --- | --- | --- | --- | --- |
|  | **coverage^a^** | **average_hit^b^** | **pc90^c^** | **coverage^a^** | **average_hit^b^** | **pc90^c^** | **coverage^a^** | **average_hit^b^** | **pc90^c^** |
| [Algeria](http://tools.iedb.org/population/result/#Algeria) | 0.0% | 0.0 | 0.0 | 77.15% | 3.98 | 1.75 | 77.15% | 3.98 | 1.75 |
|  |  |  |  |  |  |  |  |  |  |
| [Algeria Arab](http://tools.iedb.org/population/result/#Algeria%20Arab) | 0.0% | 0.0 | 0.0 | 77.15% | 3.98 | 1.75 | 77.15% | 3.98 | 1.75 |
|  |  |  |  |  |  |  |  |  |  |
| [American Samoa](http://tools.iedb.org/population/result/#American%20Samoa) | 98.75% | 8.09 | 5.53 | 0.0% | 0.0 | 0.0 | 98.75% | 8.09 | 5.53 |
|  |  |  |  |  |  |  |  |  |  |
| [American Samoa Polynesian](http://tools.iedb.org/population/result/#American%20Samoa%20Polynesian) | 98.75% | 8.09 | 5.53 | 0.0% | 0.0 | 0.0 | 98.75% | 8.09 | 5.53 |
|  |  |  |  |  |  |  |  |  |  |
| [Argentina](http://tools.iedb.org/population/result/#Argentina) | 97.5% | 7.07 | 4.88 | 62.67% | 3.03 | 1.07 | 99.07% | 10.1 | 6.61 |
|  |  |  |  |  |  |  |  |  |  |
| [Argentina Amerindian](http://tools.iedb.org/population/result/#Argentina%20Amerindian) | 97.5% | 7.07 | 4.88 | 45.78% | 2.02 | 0.74 | 98.64% | 9.09 | 5.78 |
|  |  |  |  |  |  |  |  |  |  |
| [Argentina Caucasoid](http://tools.iedb.org/population/result/#Argentina%20Caucasoid) | 0.0% | 0.0 | 0.0 | 80.65% | 4.3 | 2.07 | 80.65% | 4.3 | 2.07 |
|  |  |  |  |  |  |  |  |  |  |
| [Australia](http://tools.iedb.org/population/result/#Australia) | 94.51% | 7.33 | 4.59 | 33.15% | 1.43 | 0.6 | 96.33% | 8.77 | 5.14 |
|  |  |  |  |  |  |  |  |  |  |
| [Australia Australian Aborigines](http://tools.iedb.org/population/result/#Australia%20Australian%20Aborigines) | 87.31% | 5.42 | 3.15 | 33.15% | 1.43 | 0.6 | 91.52% | 6.86 | 4.17 |
|  |  |  |  |  |  |  |  |  |  |
| [Australia Caucasoid](http://tools.iedb.org/population/result/#Australia%20Caucasoid) | 99.94% | 12.18 | 9.42 | 0.0% | 0.0 | 0.0 | 99.94% | 12.18 | 9.42 |
|  |  |  |  |  |  |  |  |  |  |
| [Austria](http://tools.iedb.org/population/result/#Austria) | 99.91% | 11.46 | 8.8 | 93.34% | 5.62 | 4.29 | 99.99% | 17.08 | 13.31 |
|  |  |  |  |  |  |  |  |  |  |
| [Austria Caucasoid](http://tools.iedb.org/population/result/#Austria%20Caucasoid) | 99.91% | 11.46 | 8.8 | 93.34% | 5.62 | 4.29 | 99.99% | 17.08 | 13.31 |
|  |  |  |  |  |  |  |  |  |  |
| [Belarus](http://tools.iedb.org/population/result/#Belarus) | 0.0% | 0.0 | 0.0 | 43.81% | 1.87 | 0.71 | 43.81% | 1.87 | 0.71 |
|  |  |  |  |  |  |  |  |  |  |
| [Belarus Caucasoid](http://tools.iedb.org/population/result/#Belarus%20Caucasoid) | 0.0% | 0.0 | 0.0 | 43.81% | 1.87 | 0.71 | 43.81% | 1.87 | 0.71 |
|  |  |  |  |  |  |  |  |  |  |
| [Belgium](http://tools.iedb.org/population/result/#Belgium) | 99.39% | 8.88 | 6.22 | 79.39% | 4.15 | 1.94 | 99.87% | 13.03 | 9.27 |
|  |  |  |  |  |  |  |  |  |  |
| [Belgium Caucasoid](http://tools.iedb.org/population/result/#Belgium%20Caucasoid) | 99.39% | 8.88 | 6.22 | 79.39% | 4.15 | 1.94 | 99.87% | 13.03 | 9.27 |
|  |  |  |  |  |  |  |  |  |  |
| [Bolivia](http://tools.iedb.org/population/result/#Bolivia) | 0.0% | 0.0 | 0.0 | 77.82% | 3.72 | 1.8 | 77.82% | 3.72 | 1.8 |
|  |  |  |  |  |  |  |  |  |  |
| [Bolivia Amerindian](http://tools.iedb.org/population/result/#Bolivia%20Amerindian) | 0.0% | 0.0 | 0.0 | 77.82% | 3.72 | 1.8 | 77.82% | 3.72 | 1.8 |
|  |  |  |  |  |  |  |  |  |  |
| [Borneo](http://tools.iedb.org/population/result/#Borneo) | 0.0% | 0.0 | 0.0 | 49.02% | 2.16 | 0.78 | 49.02% | 2.16 | 0.78 |
|  |  |  |  |  |  |  |  |  |  |
| [Borneo Austronesian](http://tools.iedb.org/population/result/#Borneo%20Austronesian) | 0.0% | 0.0 | 0.0 | 49.02% | 2.16 | 0.78 | 49.02% | 2.16 | 0.78 |
|  |  |  |  |  |  |  |  |  |  |
| [Brazil](http://tools.iedb.org/population/result/#Brazil) | 96.1% | 7.81 | 4.92 | 63.8% | 3.07 | 1.1 | 98.59% | 10.88 | 6.95 |
|  |  |  |  |  |  |  |  |  |  |
| [Brazil Amerindian](http://tools.iedb.org/population/result/#Brazil%20Amerindian) | 93.24% | 5.49 | 4.25 | 48.6% | 2.06 | 0.78 | 96.52% | 7.55 | 4.89 |
|  |  |  |  |  |  |  |  |  |  |
| [Brazil Caucasoid](http://tools.iedb.org/population/result/#Brazil%20Caucasoid) | 99.32% | 9.83 | 7.06 | 84.39% | 4.65 | 2.56 | 99.89% | 14.48 | 10.42 |
|  |  |  |  |  |  |  |  |  |  |
| [Brazil Mixed](http://tools.iedb.org/population/result/#Brazil%20Mixed) | 97.02% | 8.55 | 5.32 | 77.5% | 4.04 | 1.78 | 99.33% | 12.59 | 8.55 |
|  |  |  |  |  |  |  |  |  |  |
| [Brazil Mulatto](http://tools.iedb.org/population/result/#Brazil%20Mulatto) | 0.0% | 0.0 | 0.0 | 74.09% | 3.8 | 1.54 | 74.09% | 3.8 | 1.54 |
|  |  |  |  |  |  |  |  |  |  |
| [Bulgaria](http://tools.iedb.org/population/result/#Bulgaria) | 99.42% | 9.47 | 6.8 | 57.23% | 2.68 | 0.94 | 99.75% | 12.15 | 8.61 |
|  |  |  |  |  |  |  |  |  |  |
| [Bulgaria Caucasoid](http://tools.iedb.org/population/result/#Bulgaria%20Caucasoid) | 99.52% | 9.6 | 7.02 | 57.23% | 2.68 | 0.94 | 99.79% | 12.28 | 8.71 |
|  |  |  |  |  |  |  |  |  |  |
| [Bulgaria Other](http://tools.iedb.org/population/result/#Bulgaria%20Other) | 99.58% | 8.26 | 5.74 | 0.0% | 0.0 | 0.0 | 99.58% | 8.26 | 5.74 |
|  |  |  |  |  |  |  |  |  |  |
| [Burkina Faso](http://tools.iedb.org/population/result/#Burkina%20Faso) | 67.18% | 3.7 | 1.22 | 0.0% | 0.0 | 0.0 | 67.18% | 3.7 | 1.22 |
|  |  |  |  |  |  |  |  |  |  |
| [Burkina Faso Black](http://tools.iedb.org/population/result/#Burkina%20Faso%20Black) | 67.18% | 3.7 | 1.22 | 0.0% | 0.0 | 0.0 | 67.18% | 3.7 | 1.22 |
|  |  |  |  |  |  |  |  |  |  |
| [Cameroon](http://tools.iedb.org/population/result/#Cameroon) | 89.72% | 6.66 | 3.89 | 49.87% | 2.27 | 0.8 | 94.85% | 8.93 | 4.91 |
|  |  |  |  |  |  |  |  |  |  |
| [Cameroon Black](http://tools.iedb.org/population/result/#Cameroon%20Black) | 89.72% | 6.66 | 3.89 | 49.87% | 2.27 | 0.8 | 94.85% | 8.93 | 4.91 |
|  |  |  |  |  |  |  |  |  |  |
| [Canada](http://tools.iedb.org/population/result/#Canada) | 0.0% | 0.0 | 0.0 | 38.41% | 1.68 | 0.65 | 38.41% | 1.68 | 0.65 |
|  |  |  |  |  |  |  |  |  |  |
| [Canada Amerindian](http://tools.iedb.org/population/result/#Canada%20Amerindian) | 0.0% | 0.0 | 0.0 | 38.41% | 1.68 | 0.65 | 38.41% | 1.68 | 0.65 |
|  |  |  |  |  |  |  |  |  |  |
| [Cape Verde](http://tools.iedb.org/population/result/#Cape%20Verde) | 99.18% | 10.35 | 7.66 | 80.38% | 4.3 | 2.04 | 99.84% | 14.65 | 10.45 |
|  |  |  |  |  |  |  |  |  |  |
| [Cape Verde Black](http://tools.iedb.org/population/result/#Cape%20Verde%20Black) | 99.18% | 10.35 | 7.66 | 80.38% | 4.3 | 2.04 | 99.84% | 14.65 | 10.45 |
|  |  |  |  |  |  |  |  |  |  |
| [Central Africa](http://tools.iedb.org/population/result/#Central%20Africa) | 86.04% | 6.0 | 2.86 | 62.71% | 3.01 | 1.07 | 94.79% | 9.01 | 4.91 |
|  |  |  |  |  |  |  |  |  |  |
| [Central African Republic](http://tools.iedb.org/population/result/#Central%20African%20Republic) | 28.02% | 1.22 | 0.56 | 82.69% | 4.28 | 2.31 | 87.54% | 5.5 | 3.21 |
|  |  |  |  |  |  |  |  |  |  |
| [Central African Republic Black](http://tools.iedb.org/population/result/#Central%20African%20Republic%20Black) | 28.02% | 1.22 | 0.56 | 82.69% | 4.28 | 2.31 | 87.54% | 5.5 | 3.21 |
|  |  |  |  |  |  |  |  |  |  |
| [Central America](http://tools.iedb.org/population/result/#Central%20America) | 7.76% | 0.32 | 0.43 | 49.91% | 2.24 | 0.8 | 53.8% | 2.56 | 0.87 |
|  |  |  |  |  |  |  |  |  |  |
| [Chile](http://tools.iedb.org/population/result/#Chile) | 95.94% | 7.63 | 4.85 | 67.08% | 3.33 | 1.21 | 98.66% | 10.96 | 7.06 |
|  |  |  |  |  |  |  |  |  |  |
| [Chile Amerindian](http://tools.iedb.org/population/result/#Chile%20Amerindian) | 99.63% | 8.75 | 5.99 | 72.65% | 3.51 | 1.46 | 99.9% | 12.26 | 8.74 |
|  |  |  |  |  |  |  |  |  |  |
| [Chile Mixed](http://tools.iedb.org/population/result/#Chile%20Mixed) | 90.09% | 6.42 | 4.01 | 52.65% | 2.44 | 0.84 | 95.31% | 8.86 | 4.97 |
|  |  |  |  |  |  |  |  |  |  |
| [China](http://tools.iedb.org/population/result/#China) | 94.57% | 7.22 | 4.57 | 59.99% | 2.87 | 1.0 | 97.83% | 10.09 | 6.04 |
|  |  |  |  |  |  |  |  |  |  |
| [China Oriental](http://tools.iedb.org/population/result/#China%20Oriental) | 94.57% | 7.22 | 4.57 | 59.99% | 2.87 | 1.0 | 97.83% | 10.09 | 6.04 |
|  |  |  |  |  |  |  |  |  |  |
| [Colombia](http://tools.iedb.org/population/result/#Colombia) | 8.36% | 0.33 | 0.44 | 54.02% | 2.51 | 0.87 | 57.86% | 2.84 | 0.95 |
|  |  |  |  |  |  |  |  |  |  |
| [Colombia Amerindian](http://tools.iedb.org/population/result/#Colombia%20Amerindian) | 0.0% | 0.0 | 0.0 | 47.4% | 2.12 | 0.76 | 47.4% | 2.12 | 0.76 |
|  |  |  |  |  |  |  |  |  |  |
| [Colombia Black](http://tools.iedb.org/population/result/#Colombia%20Black) | 3.65% | 0.15 | 0.42 | 65.25% | 3.21 | 1.15 | 66.51% | 3.35 | 1.19 |
|  |  |  |  |  |  |  |  |  |  |
| [Colombia Mestizo](http://tools.iedb.org/population/result/#Colombia%20Mestizo) | 14.07% | 0.56 | 0.47 | 56.31% | 2.62 | 0.92 | 62.45% | 3.19 | 1.07 |
|  |  |  |  |  |  |  |  |  |  |
| [Congo](http://tools.iedb.org/population/result/#Congo) | 0.0% | 0.0 | 0.0 | 68.66% | 3.39 | 1.28 | 68.66% | 3.39 | 1.28 |
|  |  |  |  |  |  |  |  |  |  |
| [Congo Black](http://tools.iedb.org/population/result/#Congo%20Black) | 0.0% | 0.0 | 0.0 | 68.66% | 3.39 | 1.28 | 68.66% | 3.39 | 1.28 |
|  |  |  |  |  |  |  |  |  |  |
| [Cook Islands](http://tools.iedb.org/population/result/#Cook%20Islands) | 0.0% | 0.0 | 0.0 | 78.59% | 4.06 | 1.87 | 78.59% | 4.06 | 1.87 |
|  |  |  |  |  |  |  |  |  |  |
| [Cook Islands Polynesian](http://tools.iedb.org/population/result/#Cook%20Islands%20Polynesian) | 0.0% | 0.0 | 0.0 | 78.59% | 4.06 | 1.87 | 78.59% | 4.06 | 1.87 |
|  |  |  |  |  |  |  |  |  |  |
| [Costa Rica](http://tools.iedb.org/population/result/#Costa%20Rica) | 0.0% | 0.0 | 0.0 | 24.31% | 1.02 | 0.53 | 24.31% | 1.02 | 0.53 |
|  |  |  |  |  |  |  |  |  |  |
| [Costa Rica Mestizo](http://tools.iedb.org/population/result/#Costa%20Rica%20Mestizo) | 0.0% | 0.0 | 0.0 | 24.31% | 1.02 | 0.53 | 24.31% | 1.02 | 0.53 |
|  |  |  |  |  |  |  |  |  |  |
| [Croatia](http://tools.iedb.org/population/result/#Croatia) | 99.79% | 10.61 | 8.24 | 66.71% | 3.26 | 1.2 | 99.93% | 13.87 | 9.91 |
|  |  |  |  |  |  |  |  |  |  |
| [Croatia Caucasoid](http://tools.iedb.org/population/result/#Croatia%20Caucasoid) | 99.79% | 10.61 | 8.24 | 66.71% | 3.26 | 1.2 | 99.93% | 13.87 | 9.91 |
|  |  |  |  |  |  |  |  |  |  |
| [Cuba](http://tools.iedb.org/population/result/#Cuba) | 98.92% | 9.96 | 6.92 | 85.48% | 4.59 | 2.76 | 99.84% | 14.55 | 10.41 |
|  |  |  |  |  |  |  |  |  |  |
| [Cuba Caucasoid](http://tools.iedb.org/population/result/#Cuba%20Caucasoid) | 99.05% | 9.9 | 6.95 | 0.0% | 0.0 | 0.0 | 99.05% | 9.9 | 6.95 |
|  |  |  |  |  |  |  |  |  |  |
| [Cuba Mixed](http://tools.iedb.org/population/result/#Cuba%20Mixed) | 0.0% | 0.0 | 0.0 | 85.48% | 4.59 | 2.76 | 85.48% | 4.59 | 2.76 |
|  |  |  |  |  |  |  |  |  |  |
| [Cuba Mulatto](http://tools.iedb.org/population/result/#Cuba%20Mulatto) | 98.74% | 9.98 | 6.84 | 0.0% | 0.0 | 0.0 | 98.74% | 9.98 | 6.84 |
|  |  |  |  |  |  |  |  |  |  |
| [Czech Republic](http://tools.iedb.org/population/result/#Czech%20Republic) | 99.08% | 9.99 | 7.03 | 86.21% | 4.78 | 2.9 | 99.87% | 14.77 | 10.65 |
|  |  |  |  |  |  |  |  |  |  |
| [Czech Republic Caucasoid](http://tools.iedb.org/population/result/#Czech%20Republic%20Caucasoid) | 99.08% | 9.99 | 7.03 | 88.76% | 5.04 | 3.56 | 99.9% | 15.03 | 11.05 |
|  |  |  |  |  |  |  |  |  |  |
| [Czech Republic Other](http://tools.iedb.org/population/result/#Czech%20Republic%20Other) | 0.0% | 0.0 | 0.0 | 64.14% | 3.05 | 1.12 | 64.14% | 3.05 | 1.12 |
|  |  |  |  |  |  |  |  |  |  |
| [Denmark](http://tools.iedb.org/population/result/#Denmark) | 0.0% | 0.0 | 0.0 | 88.98% | 4.97 | 3.63 | 88.98% | 4.97 | 3.63 |
|  |  |  |  |  |  |  |  |  |  |
| [Denmark Caucasoid](http://tools.iedb.org/population/result/#Denmark%20Caucasoid) | 0.0% | 0.0 | 0.0 | 88.98% | 4.97 | 3.63 | 88.98% | 4.97 | 3.63 |
|  |  |  |  |  |  |  |  |  |  |
| [East Africa](http://tools.iedb.org/population/result/#East%20Africa) | 90.78% | 6.7 | 4.09 | 68.3% | 3.35 | 1.26 | 97.08% | 10.06 | 5.8 |
|  |  |  |  |  |  |  |  |  |  |
| [East Asia](http://tools.iedb.org/population/result/#East%20Asia) | 98.18% | 8.92 | 5.74 | 81.82% | 4.43 | 2.2 | 99.67% | 13.35 | 9.18 |
|  |  |  |  |  |  |  |  |  |  |
| [Ecuador](http://tools.iedb.org/population/result/#Ecuador) | 77.35% | 3.53 | 1.77 | 52.17% | 2.26 | 0.84 | 89.16% | 5.78 | 3.69 |
|  |  |  |  |  |  |  |  |  |  |
| [Ecuador Amerindian](http://tools.iedb.org/population/result/#Ecuador%20Amerindian) | 77.35% | 3.53 | 1.77 | 52.17% | 2.26 | 0.84 | 89.16% | 5.78 | 3.69 |
|  |  |  |  |  |  |  |  |  |  |
| [England](http://tools.iedb.org/population/result/#England) | 99.93% | 12.03 | 9.26 | 93.48% | 5.62 | 4.3 | 100.0% | 17.65 | 13.89 |
|  |  |  |  |  |  |  |  |  |  |
| [England Caucasoid](http://tools.iedb.org/population/result/#England%20Caucasoid) | 99.93% | 12.03 | 9.26 | 93.48% | 5.62 | 4.3 | 100.0% | 17.65 | 13.89 |
|  |  |  |  |  |  |  |  |  |  |
| [Equatorial Guinea](http://tools.iedb.org/population/result/#Equatorial%20Guinea) | 0.0% | 0.0 | 0.0 | 47.58% | 2.15 | 0.76 | 47.58% | 2.15 | 0.76 |
|  |  |  |  |  |  |  |  |  |  |
| [Equatorial Guinea Black](http://tools.iedb.org/population/result/#Equatorial%20Guinea%20Black) | 0.0% | 0.0 | 0.0 | 47.58% | 2.15 | 0.76 | 47.58% | 2.15 | 0.76 |
|  |  |  |  |  |  |  |  |  |  |
| [Ethiopia](http://tools.iedb.org/population/result/#Ethiopia) | 0.0% | 0.0 | 0.0 | 83.0% | 4.36 | 2.35 | 83.0% | 4.36 | 2.35 |
|  |  |  |  |  |  |  |  |  |  |
| [Ethiopia Black](http://tools.iedb.org/population/result/#Ethiopia%20Black) | 0.0% | 0.0 | 0.0 | 83.0% | 4.36 | 2.35 | 83.0% | 4.36 | 2.35 |
|  |  |  |  |  |  |  |  |  |  |
| [Europe](http://tools.iedb.org/population/result/#Europe) | 99.68% | 10.73 | 8.23 | 85.83% | 4.77 | 2.82 | 99.96% | 15.5 | 11.96 |
|  |  |  |  |  |  |  |  |  |  |
| [Fiji](http://tools.iedb.org/population/result/#Fiji) | 0.0% | 0.0 | 0.0 | 79.87% | 3.93 | 1.99 | 79.87% | 3.93 | 1.99 |
|  |  |  |  |  |  |  |  |  |  |
| [Fiji Melanesian](http://tools.iedb.org/population/result/#Fiji%20Melanesian) | 0.0% | 0.0 | 0.0 | 79.87% | 3.93 | 1.99 | 79.87% | 3.93 | 1.99 |
|  |  |  |  |  |  |  |  |  |  |
| [Finland](http://tools.iedb.org/population/result/#Finland) | 99.99% | 12.25 | 9.55 | 51.14% | 2.33 | 0.82 | 100.0% | 14.59 | 11.32 |
|  |  |  |  |  |  |  |  |  |  |
| [Finland Caucasoid](http://tools.iedb.org/population/result/#Finland%20Caucasoid) | 99.99% | 12.25 | 9.55 | 51.14% | 2.33 | 0.82 | 100.0% | 14.59 | 11.32 |
|  |  |  |  |  |  |  |  |  |  |
| [France](http://tools.iedb.org/population/result/#France) | 99.8% | 11.02 | 8.45 | 88.54% | 5.04 | 3.49 | 99.98% | 16.06 | 12.41 |
|  |  |  |  |  |  |  |  |  |  |
| [France Caucasoid](http://tools.iedb.org/population/result/#France%20Caucasoid) | 99.8% | 11.02 | 8.45 | 88.54% | 5.04 | 3.49 | 99.98% | 16.06 | 12.41 |
|  |  |  |  |  |  |  |  |  |  |
| [Gabon](http://tools.iedb.org/population/result/#Gabon) | 0.0% | 0.0 | 0.0 | 41.78% | 1.8 | 0.69 | 41.78% | 1.8 | 0.69 |
|  |  |  |  |  |  |  |  |  |  |
| [Gabon Black](http://tools.iedb.org/population/result/#Gabon%20Black) | 0.0% | 0.0 | 0.0 | 41.78% | 1.8 | 0.69 | 41.78% | 1.8 | 0.69 |
|  |  |  |  |  |  |  |  |  |  |
| [Georgia](http://tools.iedb.org/population/result/#Georgia) | 98.32% | 9.03 | 5.88 | 75.05% | 3.8 | 1.6 | 99.58% | 12.83 | 8.84 |
|  |  |  |  |  |  |  |  |  |  |
| [Georgia Caucasoid](http://tools.iedb.org/population/result/#Georgia%20Caucasoid) | 98.94% | 9.46 | 6.44 | 75.05% | 3.8 | 1.6 | 99.74% | 13.26 | 9.21 |
|  |  |  |  |  |  |  |  |  |  |
| [Georgia Kurd](http://tools.iedb.org/population/result/#Georgia%20Kurd) | 98.19% | 8.92 | 5.83 | 0.0% | 0.0 | 0.0 | 98.19% | 8.92 | 5.83 |
|  |  |  |  |  |  |  |  |  |  |
| [Germany](http://tools.iedb.org/population/result/#Germany) | 99.93% | 11.81 | 9.06 | 91.14% | 5.33 | 4.09 | 99.99% | 17.14 | 13.33 |
|  |  |  |  |  |  |  |  |  |  |
| [Germany Caucasoid](http://tools.iedb.org/population/result/#Germany%20Caucasoid) | 99.93% | 11.81 | 9.06 | 91.14% | 5.33 | 4.09 | 99.99% | 17.14 | 13.33 |
|  |  |  |  |  |  |  |  |  |  |
| [Greece](http://tools.iedb.org/population/result/#Greece) | 0.0% | 0.0 | 0.0 | 66.92% | 3.29 | 1.21 | 66.92% | 3.29 | 1.21 |
|  |  |  |  |  |  |  |  |  |  |
| [Greece Caucasoid](http://tools.iedb.org/population/result/#Greece%20Caucasoid) | 0.0% | 0.0 | 0.0 | 66.92% | 3.29 | 1.21 | 66.92% | 3.29 | 1.21 |
|  |  |  |  |  |  |  |  |  |  |
| [Guatemala](http://tools.iedb.org/population/result/#Guatemala) | 7.76% | 0.32 | 0.43 | 49.16% | 2.15 | 0.79 | 53.11% | 2.47 | 0.85 |
|  |  |  |  |  |  |  |  |  |  |
| [Guatemala Amerindian](http://tools.iedb.org/population/result/#Guatemala%20Amerindian) | 7.76% | 0.32 | 0.43 | 49.16% | 2.15 | 0.79 | 53.11% | 2.47 | 0.85 |
|  |  |  |  |  |  |  |  |  |  |
| [Guinea-Bissau](http://tools.iedb.org/population/result/#Guinea-Bissau) | 96.39% | 8.5 | 5.19 | 71.16% | 3.56 | 1.39 | 98.96% | 12.06 | 8.14 |
|  |  |  |  |  |  |  |  |  |  |
| [Guinea-Bissau Black](http://tools.iedb.org/population/result/#Guinea-Bissau%20Black) | 96.39% | 8.5 | 5.19 | 71.16% | 3.56 | 1.39 | 98.96% | 12.06 | 8.14 |
|  |  |  |  |  |  |  |  |  |  |
| [Hong Kong](http://tools.iedb.org/population/result/#Hong%20Kong) | 96.05% | 7.63 | 4.85 | 0.0% | 0.0 | 0.0 | 96.05% | 7.63 | 4.85 |
|  |  |  |  |  |  |  |  |  |  |
| [Hong Kong Oriental](http://tools.iedb.org/population/result/#Hong%20Kong%20Oriental) | 96.05% | 7.63 | 4.85 | 0.0% | 0.0 | 0.0 | 96.05% | 7.63 | 4.85 |
|  |  |  |  |  |  |  |  |  |  |
| [India](http://tools.iedb.org/population/result/#India) | 89.41% | 6.32 | 3.78 | 74.99% | 3.82 | 1.6 | 97.35% | 10.14 | 5.95 |
|  |  |  |  |  |  |  |  |  |  |
| [India Asian](http://tools.iedb.org/population/result/#India%20Asian) | 89.41% | 6.32 | 3.78 | 74.99% | 3.82 | 1.6 | 97.35% | 10.14 | 5.95 |
|  |  |  |  |  |  |  |  |  |  |
| [Indonesia](http://tools.iedb.org/population/result/#Indonesia) | 86.79% | 5.84 | 3.03 | 47.84% | 2.16 | 0.77 | 93.11% | 7.99 | 4.47 |
|  |  |  |  |  |  |  |  |  |  |
| [Indonesia Austronesian](http://tools.iedb.org/population/result/#Indonesia%20Austronesian) | 86.79% | 5.84 | 3.03 | 47.84% | 2.16 | 0.77 | 93.11% | 7.99 | 4.47 |
|  |  |  |  |  |  |  |  |  |  |
| [Iran](http://tools.iedb.org/population/result/#Iran) | 97.5% | 8.75 | 5.52 | 64.22% | 3.1 | 1.12 | 99.11% | 11.85 | 8.12 |
|  |  |  |  |  |  |  |  |  |  |
| [Iran Kurd](http://tools.iedb.org/population/result/#Iran%20Kurd) | 0.0% | 0.0 | 0.0 | 55.78% | 2.59 | 0.9 | 55.78% | 2.59 | 0.9 |
|  |  |  |  |  |  |  |  |  |  |
| [Iran Persian](http://tools.iedb.org/population/result/#Iran%20Persian) | 97.5% | 8.75 | 5.52 | 65.72% | 3.19 | 1.17 | 99.14% | 11.94 | 8.17 |
|  |  |  |  |  |  |  |  |  |  |
| [Ireland Northern](http://tools.iedb.org/population/result/#Ireland%20Northern) | 99.94% | 12.24 | 9.48 | 94.65% | 5.74 | 4.4 | 100.0% | 17.97 | 14.35 |
|  |  |  |  |  |  |  |  |  |  |
| [Ireland Northern Caucasoid](http://tools.iedb.org/population/result/#Ireland%20Northern%20Caucasoid) | 99.94% | 12.24 | 9.48 | 94.65% | 5.74 | 4.4 | 100.0% | 17.97 | 14.35 |
|  |  |  |  |  |  |  |  |  |  |
| [Ireland South](http://tools.iedb.org/population/result/#Ireland%20South) | 99.93% | 12.03 | 9.29 | 93.15% | 5.5 | 4.26 | 100.0% | 17.53 | 13.8 |
|  |  |  |  |  |  |  |  |  |  |
| [Ireland South Caucasoid](http://tools.iedb.org/population/result/#Ireland%20South%20Caucasoid) | 99.93% | 12.03 | 9.29 | 93.15% | 5.5 | 4.26 | 100.0% | 17.53 | 13.8 |
|  |  |  |  |  |  |  |  |  |  |
| [Israel](http://tools.iedb.org/population/result/#Israel) | 89.39% | 6.23 | 3.77 | 68.79% | 3.38 | 1.28 | 96.69% | 9.61 | 5.53 |
|  |  |  |  |  |  |  |  |  |  |
| [Israel Arab](http://tools.iedb.org/population/result/#Israel%20Arab) | 94.42% | 7.44 | 4.6 | 67.51% | 3.3 | 1.23 | 98.19% | 10.74 | 6.62 |
|  |  |  |  |  |  |  |  |  |  |
| [Israel Jew](http://tools.iedb.org/population/result/#Israel%20Jew) | 93.75% | 6.95 | 4.45 | 69.65% | 3.43 | 1.32 | 98.1% | 10.38 | 6.39 |
|  |  |  |  |  |  |  |  |  |  |
| [Italy](http://tools.iedb.org/population/result/#Italy) | 99.03% | 10.05 | 7.13 | 85.9% | 4.37 | 2.84 | 99.86% | 14.42 | 10.45 |
|  |  |  |  |  |  |  |  |  |  |
| [Italy Caucasoid](http://tools.iedb.org/population/result/#Italy%20Caucasoid) | 99.03% | 10.05 | 7.13 | 85.9% | 4.37 | 2.84 | 99.86% | 14.42 | 10.45 |
|  |  |  |  |  |  |  |  |  |  |
| [Ivory Coast](http://tools.iedb.org/population/result/#Ivory%20Coast) | 67.75% | 3.22 | 1.24 | 0.0% | 0.0 | 0.0 | 67.75% | 3.22 | 1.24 |
|  |  |  |  |  |  |  |  |  |  |
| [Ivory Coast Black](http://tools.iedb.org/population/result/#Ivory%20Coast%20Black) | 67.75% | 3.22 | 1.24 | 0.0% | 0.0 | 0.0 | 67.75% | 3.22 | 1.24 |
|  |  |  |  |  |  |  |  |  |  |
| [Jamaica](http://tools.iedb.org/population/result/#Jamaica) | 0.0% | 0.0 | 0.0 | 27.41% | 1.16 | 0.55 | 27.41% | 1.16 | 0.55 |
|  |  |  |  |  |  |  |  |  |  |
| [Jamaica Black](http://tools.iedb.org/population/result/#Jamaica%20Black) | 0.0% | 0.0 | 0.0 | 27.41% | 1.16 | 0.55 | 27.41% | 1.16 | 0.55 |
|  |  |  |  |  |  |  |  |  |  |
| [Japan](http://tools.iedb.org/population/result/#Japan) | 98.63% | 9.12 | 6.0 | 74.83% | 3.83 | 1.59 | 99.66% | 12.95 | 8.92 |
|  |  |  |  |  |  |  |  |  |  |
| [Japan Oriental](http://tools.iedb.org/population/result/#Japan%20Oriental) | 98.63% | 9.12 | 6.0 | 74.83% | 3.83 | 1.59 | 99.66% | 12.95 | 8.92 |
|  |  |  |  |  |  |  |  |  |  |
| [Jordan](http://tools.iedb.org/population/result/#Jordan) | 90.83% | 6.29 | 4.09 | 52.88% | 2.4 | 0.85 | 95.68% | 8.69 | 5.02 |
|  |  |  |  |  |  |  |  |  |  |
| [Jordan Arab](http://tools.iedb.org/population/result/#Jordan%20Arab) | 90.83% | 6.29 | 4.09 | 52.88% | 2.4 | 0.85 | 95.68% | 8.69 | 5.02 |
|  |  |  |  |  |  |  |  |  |  |
| [Kenya](http://tools.iedb.org/population/result/#Kenya) | 89.56% | 6.45 | 3.83 | 0.0% | 0.0 | 0.0 | 89.56% | 6.45 | 3.83 |
|  |  |  |  |  |  |  |  |  |  |
| [Kenya Black](http://tools.iedb.org/population/result/#Kenya%20Black) | 89.56% | 6.45 | 3.83 | 0.0% | 0.0 | 0.0 | 89.56% | 6.45 | 3.83 |
|  |  |  |  |  |  |  |  |  |  |
| [Kiribati](http://tools.iedb.org/population/result/#Kiribati) | 0.0% | 0.0 | 0.0 | 10.89% | 0.44 | 0.45 | 10.89% | 0.44 | 0.45 |
|  |  |  |  |  |  |  |  |  |  |
| [Kiribati Micronesian](http://tools.iedb.org/population/result/#Kiribati%20Micronesian) | 0.0% | 0.0 | 0.0 | 10.89% | 0.44 | 0.45 | 10.89% | 0.44 | 0.45 |
|  |  |  |  |  |  |  |  |  |  |
| [Korea; South](http://tools.iedb.org/population/result/#Korea;%20South) | 98.21% | 9.16 | 5.92 | 85.32% | 4.76 | 2.72 | 99.74% | 13.92 | 9.65 |
|  |  |  |  |  |  |  |  |  |  |
| [Korea; South Oriental](http://tools.iedb.org/population/result/#Korea;%20South%20Oriental) | 98.21% | 9.16 | 5.92 | 85.32% | 4.76 | 2.72 | 99.74% | 13.92 | 9.65 |
|  |  |  |  |  |  |  |  |  |  |
| [Lebanon](http://tools.iedb.org/population/result/#Lebanon) | 0.0% | 0.0 | 0.0 | 70.46% | 3.49 | 1.35 | 70.46% | 3.49 | 1.35 |
|  |  |  |  |  |  |  |  |  |  |
| [Lebanon Arab](http://tools.iedb.org/population/result/#Lebanon%20Arab) | 0.0% | 0.0 | 0.0 | 70.46% | 3.49 | 1.35 | 70.46% | 3.49 | 1.35 |
|  |  |  |  |  |  |  |  |  |  |
| [Macedonia](http://tools.iedb.org/population/result/#Macedonia) | 26.72% | 1.15 | 0.55 | 66.53% | 3.26 | 1.2 | 75.47% | 4.42 | 1.63 |
|  |  |  |  |  |  |  |  |  |  |
| [Macedonia Caucasoid](http://tools.iedb.org/population/result/#Macedonia%20Caucasoid) | 26.72% | 1.15 | 0.55 | 66.53% | 3.26 | 1.2 | 75.47% | 4.42 | 1.63 |
|  |  |  |  |  |  |  |  |  |  |
| [Malaysia](http://tools.iedb.org/population/result/#Malaysia) | 81.38% | 4.95 | 2.15 | 57.99% | 2.74 | 0.95 | 92.18% | 7.68 | 4.31 |
|  |  |  |  |  |  |  |  |  |  |
| [Malaysia Austronesian](http://tools.iedb.org/population/result/#Malaysia%20Austronesian) | 63.18% | 3.36 | 1.09 | 55.38% | 2.59 | 0.9 | 83.57% | 5.95 | 2.43 |
|  |  |  |  |  |  |  |  |  |  |
| [Malaysia Oriental](http://tools.iedb.org/population/result/#Malaysia%20Oriental) | 87.82% | 5.68 | 3.28 | 70.35% | 3.44 | 1.35 | 96.39% | 9.12 | 5.3 |
|  |  |  |  |  |  |  |  |  |  |
| [Mali](http://tools.iedb.org/population/result/#Mali) | 96.02% | 8.05 | 4.98 | 0.0% | 0.0 | 0.0 | 96.02% | 8.05 | 4.98 |
|  |  |  |  |  |  |  |  |  |  |
| [Mali Black](http://tools.iedb.org/population/result/#Mali%20Black) | 96.02% | 8.05 | 4.98 | 0.0% | 0.0 | 0.0 | 96.02% | 8.05 | 4.98 |
|  |  |  |  |  |  |  |  |  |  |
| [Martinique](http://tools.iedb.org/population/result/#Martinique) | 22.56% | 0.9 | 0.52 | 74.51% | 3.84 | 1.57 | 80.26% | 4.74 | 2.03 |
|  |  |  |  |  |  |  |  |  |  |
| [Martinique Black](http://tools.iedb.org/population/result/#Martinique%20Black) | 22.56% | 0.9 | 0.52 | 74.51% | 3.84 | 1.57 | 80.26% | 4.74 | 2.03 |
|  |  |  |  |  |  |  |  |  |  |
| [Mexico](http://tools.iedb.org/population/result/#Mexico) | 97.97% | 7.98 | 5.27 | 55.04% | 2.54 | 0.89 | 99.09% | 10.52 | 6.98 |
|  |  |  |  |  |  |  |  |  |  |
| [Mexico Amerindian](http://tools.iedb.org/population/result/#Mexico%20Amerindian) | 99.87% | 8.91 | 6.6 | 42.59% | 1.84 | 0.7 | 99.93% | 10.75 | 8.13 |
|  |  |  |  |  |  |  |  |  |  |
| [Mexico Mestizo](http://tools.iedb.org/population/result/#Mexico%20Mestizo) | 98.13% | 8.32 | 5.46 | 68.51% | 3.38 | 1.27 | 99.41% | 11.7 | 8.21 |
|  |  |  |  |  |  |  |  |  |  |
| [Mongolia](http://tools.iedb.org/population/result/#Mongolia) | 95.31% | 5.9 | 4.49 | 81.85% | 4.45 | 2.2 | 99.15% | 10.35 | 7.56 |
|  |  |  |  |  |  |  |  |  |  |
| [Mongolia Oriental](http://tools.iedb.org/population/result/#Mongolia%20Oriental) | 95.31% | 5.9 | 4.49 | 81.85% | 4.45 | 2.2 | 99.15% | 10.35 | 7.56 |
|  |  |  |  |  |  |  |  |  |  |
| [Morocco](http://tools.iedb.org/population/result/#Morocco) | 98.63% | 9.3 | 6.21 | 83.44% | 4.48 | 2.41 | 99.77% | 13.77 | 9.64 |
|  |  |  |  |  |  |  |  |  |  |
| [Morocco Arab](http://tools.iedb.org/population/result/#Morocco%20Arab) | 99.32% | 9.95 | 7.3 | 85.07% | 4.62 | 2.68 | 99.9% | 14.57 | 10.62 |
|  |  |  |  |  |  |  |  |  |  |
| [Morocco Caucasoid](http://tools.iedb.org/population/result/#Morocco%20Caucasoid) | 97.94% | 8.8 | 5.66 | 79.75% | 4.15 | 1.98 | 99.58% | 12.95 | 8.93 |
|  |  |  |  |  |  |  |  |  |  |
| [Nauru](http://tools.iedb.org/population/result/#Nauru) | 0.0% | 0.0 | 0.0 | 38.66% | 1.68 | 0.65 | 38.66% | 1.68 | 0.65 |
|  |  |  |  |  |  |  |  |  |  |
| [Nauru Micronesian](http://tools.iedb.org/population/result/#Nauru%20Micronesian) | 0.0% | 0.0 | 0.0 | 38.66% | 1.68 | 0.65 | 38.66% | 1.68 | 0.65 |
|  |  |  |  |  |  |  |  |  |  |
| [Netherlands](http://tools.iedb.org/population/result/#Netherlands) | 0.0% | 0.0 | 0.0 | 83.44% | 4.55 | 2.41 | 83.44% | 4.55 | 2.41 |
|  |  |  |  |  |  |  |  |  |  |
| [Netherlands Caucasoid](http://tools.iedb.org/population/result/#Netherlands%20Caucasoid) | 0.0% | 0.0 | 0.0 | 83.44% | 4.55 | 2.41 | 83.44% | 4.55 | 2.41 |
|  |  |  |  |  |  |  |  |  |  |
| [New Caledonia](http://tools.iedb.org/population/result/#New%20Caledonia) | 98.18% | 6.59 | 4.74 | 81.41% | 4.03 | 2.15 | 99.66% | 10.61 | 8.03 |
|  |  |  |  |  |  |  |  |  |  |
| [New Caledonia Melanesian](http://tools.iedb.org/population/result/#New%20Caledonia%20Melanesian) | 98.18% | 6.59 | 4.74 | 81.41% | 4.03 | 2.15 | 99.66% | 10.61 | 8.03 |
|  |  |  |  |  |  |  |  |  |  |
| [New Zealand](http://tools.iedb.org/population/result/#New%20Zealand) | 0.0% | 0.0 | 0.0 | 84.46% | 4.56 | 2.57 | 84.46% | 4.56 | 2.57 |
|  |  |  |  |  |  |  |  |  |  |
| [New Zealand Polynesian](http://tools.iedb.org/population/result/#New%20Zealand%20Polynesian) | 0.0% | 0.0 | 0.0 | 84.46% | 4.56 | 2.57 | 84.46% | 4.56 | 2.57 |
|  |  |  |  |  |  |  |  |  |  |
| [Niue](http://tools.iedb.org/population/result/#Niue) | 0.0% | 0.0 | 0.0 | 77.82% | 3.81 | 1.8 | 77.82% | 3.81 | 1.8 |
|  |  |  |  |  |  |  |  |  |  |
| [Niue Polynesian](http://tools.iedb.org/population/result/#Niue%20Polynesian) | 0.0% | 0.0 | 0.0 | 77.82% | 3.81 | 1.8 | 77.82% | 3.81 | 1.8 |
|  |  |  |  |  |  |  |  |  |  |
| [North Africa](http://tools.iedb.org/population/result/#North%20Africa) | 96.03% | 7.97 | 4.95 | 75.06% | 3.83 | 1.6 | 99.01% | 11.81 | 8.09 |
|  |  |  |  |  |  |  |  |  |  |
| [North America](http://tools.iedb.org/population/result/#North%20America) | 99.06% | 9.79 | 6.79 | 87.89% | 4.99 | 3.3 | 99.89% | 14.78 | 10.72 |
|  |  |  |  |  |  |  |  |  |  |
| [Northeast Asia](http://tools.iedb.org/population/result/#Northeast%20Asia) | 94.7% | 7.27 | 4.6 | 59.99% | 2.87 | 1.0 | 97.88% | 10.14 | 6.08 |
|  |  |  |  |  |  |  |  |  |  |
| [Norway](http://tools.iedb.org/population/result/#Norway) | 0.0% | 0.0 | 0.0 | 94.71% | 5.79 | 4.42 | 94.71% | 5.79 | 4.42 |
|  |  |  |  |  |  |  |  |  |  |
| [Norway Caucasoid](http://tools.iedb.org/population/result/#Norway%20Caucasoid) | 0.0% | 0.0 | 0.0 | 94.71% | 5.79 | 4.42 | 94.71% | 5.79 | 4.42 |
|  |  |  |  |  |  |  |  |  |  |
| [Oceania](http://tools.iedb.org/population/result/#Oceania) | 94.71% | 6.65 | 4.49 | 59.87% | 2.84 | 1.0 | 97.88% | 9.49 | 5.75 |
|  |  |  |  |  |  |  |  |  |  |
| [Oman](http://tools.iedb.org/population/result/#Oman) | 99.69% | 10.46 | 8.17 | 0.0% | 0.0 | 0.0 | 99.69% | 10.46 | 8.17 |
|  |  |  |  |  |  |  |  |  |  |
| [Oman Arab](http://tools.iedb.org/population/result/#Oman%20Arab) | 99.69% | 10.46 | 8.17 | 0.0% | 0.0 | 0.0 | 99.69% | 10.46 | 8.17 |
|  |  |  |  |  |  |  |  |  |  |
| [Pakistan](http://tools.iedb.org/population/result/#Pakistan) | 97.09% | 6.37 | 4.81 | 1.18% | 0.05 | 0.4 | 97.13% | 6.41 | 4.82 |
|  |  |  |  |  |  |  |  |  |  |
| [Pakistan Asian](http://tools.iedb.org/population/result/#Pakistan%20Asian) | 96.75% | 6.3 | 4.75 | 1.45% | 0.06 | 0.41 | 96.79% | 6.36 | 4.77 |
|  |  |  |  |  |  |  |  |  |  |
| [Pakistan Mixed](http://tools.iedb.org/population/result/#Pakistan%20Mixed) | 97.73% | 6.46 | 4.91 | 0.0% | 0.0 | 0.4 | 97.73% | 6.46 | 4.91 |
|  |  |  |  |  |  |  |  |  |  |
| [Papua New Guinea](http://tools.iedb.org/population/result/#Papua%20New%20Guinea) | 97.92% | 5.93 | 4.6 | 69.15% | 3.36 | 1.3 | 99.36% | 9.29 | 6.16 |
|  |  |  |  |  |  |  |  |  |  |
| [Papua New Guinea Melanesian](http://tools.iedb.org/population/result/#Papua%20New%20Guinea%20Melanesian) | 97.92% | 5.93 | 4.6 | 69.15% | 3.36 | 1.3 | 99.36% | 9.29 | 6.16 |
|  |  |  |  |  |  |  |  |  |  |
| [Paraguay](http://tools.iedb.org/population/result/#Paraguay) | 0.0% | 0.0 | 0.0 | 4.9% | 0.2 | 0.42 | 4.9% | 0.2 | 0.42 |
|  |  |  |  |  |  |  |  |  |  |
| [Paraguay Amerindian](http://tools.iedb.org/population/result/#Paraguay%20Amerindian) | 0.0% | 0.0 | 0.0 | 4.9% | 0.2 | 0.42 | 4.9% | 0.2 | 0.42 |
|  |  |  |  |  |  |  |  |  |  |
| [Peru](http://tools.iedb.org/population/result/#Peru) | 99.99% | 7.86 | 5.58 | 49.87% | 2.19 | 0.8 | 100.0% | 10.05 | 7.15 |
|  |  |  |  |  |  |  |  |  |  |
| [Peru Amerindian](http://tools.iedb.org/population/result/#Peru%20Amerindian) | 99.99% | 7.86 | 5.58 | 49.87% | 2.19 | 0.8 | 100.0% | 10.05 | 7.15 |
|  |  |  |  |  |  |  |  |  |  |
| [Philippines](http://tools.iedb.org/population/result/#Philippines) | 94.98% | 6.29 | 4.46 | 28.56% | 1.21 | 0.56 | 96.41% | 7.5 | 4.8 |
|  |  |  |  |  |  |  |  |  |  |
| [Philippines Austronesian](http://tools.iedb.org/population/result/#Philippines%20Austronesian) | 94.98% | 6.29 | 4.46 | 28.56% | 1.21 | 0.56 | 96.41% | 7.5 | 4.8 |
|  |  |  |  |  |  |  |  |  |  |
| [Poland](http://tools.iedb.org/population/result/#Poland) | 99.77% | 10.75 | 8.28 | 84.46% | 4.62 | 2.57 | 99.96% | 15.37 | 11.82 |
|  |  |  |  |  |  |  |  |  |  |
| [Poland Caucasoid](http://tools.iedb.org/population/result/#Poland%20Caucasoid) | 99.77% | 10.75 | 8.28 | 84.46% | 4.62 | 2.57 | 99.96% | 15.37 | 11.82 |
|  |  |  |  |  |  |  |  |  |  |
| [Portugal](http://tools.iedb.org/population/result/#Portugal) | 98.72% | 9.48 | 6.34 | 78.0% | 4.06 | 1.82 | 99.72% | 13.54 | 9.35 |
|  |  |  |  |  |  |  |  |  |  |
| [Portugal Caucasoid](http://tools.iedb.org/population/result/#Portugal%20Caucasoid) | 98.72% | 9.48 | 6.34 | 78.0% | 4.06 | 1.82 | 99.72% | 13.54 | 9.35 |
|  |  |  |  |  |  |  |  |  |  |
| [Romania](http://tools.iedb.org/population/result/#Romania) | 99.67% | 10.27 | 8.03 | 0.0% | 0.0 | 0.0 | 99.67% | 10.27 | 8.03 |
|  |  |  |  |  |  |  |  |  |  |
| [Romania Caucasoid](http://tools.iedb.org/population/result/#Romania%20Caucasoid) | 99.67% | 10.27 | 8.03 | 0.0% | 0.0 | 0.0 | 99.67% | 10.27 | 8.03 |
|  |  |  |  |  |  |  |  |  |  |
| [Russia](http://tools.iedb.org/population/result/#Russia) | 99.27% | 9.57 | 6.76 | 77.62% | 4.09 | 1.79 | 99.84% | 13.66 | 9.57 |
|  |  |  |  |  |  |  |  |  |  |
| [Russia Caucasoid](http://tools.iedb.org/population/result/#Russia%20Caucasoid) | 3.96% | 0.16 | 0.42 | 88.52% | 5.06 | 3.48 | 88.97% | 5.22 | 3.63 |
|  |  |  |  |  |  |  |  |  |  |
| [Russia Mixed](http://tools.iedb.org/population/result/#Russia%20Mixed) | 5.05% | 0.2 | 0.42 | 0.0% | 0.0 | 0.0 | 5.05% | 0.2 | 0.42 |
|  |  |  |  |  |  |  |  |  |  |
| [Russia Other](http://tools.iedb.org/population/result/#Russia%20Other) | 99.98% | 10.06 | 8.19 | 85.01% | 4.64 | 2.67 | 100.0% | 14.7 | 11.33 |
|  |  |  |  |  |  |  |  |  |  |
| [Russia Siberian](http://tools.iedb.org/population/result/#Russia%20Siberian) | 99.43% | 10.19 | 7.6 | 78.83% | 4.18 | 1.89 | 99.88% | 14.36 | 10.21 |
|  |  |  |  |  |  |  |  |  |  |
| [Rwanda](http://tools.iedb.org/population/result/#Rwanda) | 24.87% | 1.07 | 0.53 | 62.79% | 2.99 | 1.07 | 72.05% | 4.06 | 1.43 |
|  |  |  |  |  |  |  |  |  |  |
| [Rwanda Black](http://tools.iedb.org/population/result/#Rwanda%20Black) | 24.87% | 1.07 | 0.53 | 62.79% | 2.99 | 1.07 | 72.05% | 4.06 | 1.43 |
|  |  |  |  |  |  |  |  |  |  |
| [Samoa](http://tools.iedb.org/population/result/#Samoa) | 0.0% | 0.0 | 0.0 | 80.86% | 4.24 | 2.09 | 80.86% | 4.24 | 2.09 |
|  |  |  |  |  |  |  |  |  |  |
| [Samoa Polynesian](http://tools.iedb.org/population/result/#Samoa%20Polynesian) | 0.0% | 0.0 | 0.0 | 80.86% | 4.24 | 2.09 | 80.86% | 4.24 | 2.09 |
|  |  |  |  |  |  |  |  |  |  |
| [Sao Tome and Principe](http://tools.iedb.org/population/result/#Sao%20Tome%20and%20Principe) | 97.02% | 8.87 | 5.49 | 66.5% | 3.24 | 1.19 | 99.0% | 12.11 | 8.19 |
|  |  |  |  |  |  |  |  |  |  |
| [Sao Tome and Principe Black](http://tools.iedb.org/population/result/#Sao%20Tome%20and%20Principe%20Black) | 97.02% | 8.87 | 5.49 | 66.5% | 3.24 | 1.19 | 99.0% | 12.11 | 8.19 |
|  |  |  |  |  |  |  |  |  |  |
| [Saudi Arabia](http://tools.iedb.org/population/result/#Saudi%20Arabia) | 98.26% | 8.81 | 5.78 | 80.14% | 4.19 | 2.01 | 99.65% | 13.0 | 9.04 |
|  |  |  |  |  |  |  |  |  |  |
| [Saudi Arabia Arab](http://tools.iedb.org/population/result/#Saudi%20Arabia%20Arab) | 98.26% | 8.81 | 5.78 | 80.14% | 4.19 | 2.01 | 99.65% | 13.0 | 9.04 |
|  |  |  |  |  |  |  |  |  |  |
| [Scotland](http://tools.iedb.org/population/result/#Scotland) | 65.34% | 3.5 | 1.15 | 90.82% | 5.25 | 4.07 | 96.82% | 8.75 | 5.26 |
|  |  |  |  |  |  |  |  |  |  |
| [Scotland Caucasoid](http://tools.iedb.org/population/result/#Scotland%20Caucasoid) | 65.34% | 3.5 | 1.15 | 90.82% | 5.25 | 4.07 | 96.82% | 8.75 | 5.26 |
|  |  |  |  |  |  |  |  |  |  |
| [Senegal](http://tools.iedb.org/population/result/#Senegal) | 95.58% | 7.92 | 4.87 | 30.28% | 1.3 | 0.57 | 96.92% | 9.22 | 5.44 |
|  |  |  |  |  |  |  |  |  |  |
| [Senegal Black](http://tools.iedb.org/population/result/#Senegal%20Black) | 95.58% | 7.92 | 4.87 | 30.28% | 1.3 | 0.57 | 96.92% | 9.22 | 5.44 |
|  |  |  |  |  |  |  |  |  |  |
| [Serbia](http://tools.iedb.org/population/result/#Serbia) | 73.37% | 3.73 | 1.5 | 0.0% | 0.0 | 0.0 | 73.37% | 3.73 | 1.5 |
|  |  |  |  |  |  |  |  |  |  |
| [Serbia Caucasoid](http://tools.iedb.org/population/result/#Serbia%20Caucasoid) | 73.37% | 3.73 | 1.5 | 0.0% | 0.0 | 0.0 | 73.37% | 3.73 | 1.5 |
|  |  |  |  |  |  |  |  |  |  |
| [Singapore](http://tools.iedb.org/population/result/#Singapore) | 92.66% | 6.84 | 4.31 | 65.78% | 3.2 | 1.17 | 97.49% | 10.04 | 5.92 |
|  |  |  |  |  |  |  |  |  |  |
| [Singapore Austronesian](http://tools.iedb.org/population/result/#Singapore%20Austronesian) | 90.55% | 6.48 | 4.06 | 65.78% | 3.2 | 1.17 | 96.77% | 9.68 | 5.55 |
|  |  |  |  |  |  |  |  |  |  |
| [Singapore Oriental](http://tools.iedb.org/population/result/#Singapore%20Oriental) | 94.81% | 7.35 | 4.63 | 0.0% | 0.0 | 0.0 | 94.81% | 7.35 | 4.63 |
|  |  |  |  |  |  |  |  |  |  |
| [Slovakia](http://tools.iedb.org/population/result/#Slovakia) | 0.0% | 0.0 | 0.0 | 18.28% | 0.73 | 0.49 | 18.28% | 0.73 | 0.49 |
|  |  |  |  |  |  |  |  |  |  |
| [Slovakia Caucasoid](http://tools.iedb.org/population/result/#Slovakia%20Caucasoid) | 0.0% | 0.0 | 0.0 | 18.28% | 0.73 | 0.49 | 18.28% | 0.73 | 0.49 |
|  |  |  |  |  |  |  |  |  |  |
| [Slovenia](http://tools.iedb.org/population/result/#Slovenia) | 0.0% | 0.0 | 0.0 | 84.85% | 4.66 | 2.64 | 84.85% | 4.66 | 2.64 |
|  |  |  |  |  |  |  |  |  |  |
| [Slovenia Caucasoid](http://tools.iedb.org/population/result/#Slovenia%20Caucasoid) | 0.0% | 0.0 | 0.0 | 84.85% | 4.66 | 2.64 | 84.85% | 4.66 | 2.64 |
|  |  |  |  |  |  |  |  |  |  |
| [South Africa](http://tools.iedb.org/population/result/#South%20Africa) | 93.03% | 7.14 | 4.39 | 32.1% | 1.33 | 0.59 | 95.27% | 8.47 | 4.9 |
|  |  |  |  |  |  |  |  |  |  |
| [South Africa Black](http://tools.iedb.org/population/result/#South%20Africa%20Black) | 91.96% | 6.98 | 4.24 | 32.1% | 1.33 | 0.59 | 94.54% | 8.31 | 4.74 |
|  |  |  |  |  |  |  |  |  |  |
| [South Africa Other](http://tools.iedb.org/population/result/#South%20Africa%20Other) | 97.61% | 8.42 | 5.38 | 0.0% | 0.0 | 0.0 | 97.61% | 8.42 | 5.38 |
|  |  |  |  |  |  |  |  |  |  |
| [South America](http://tools.iedb.org/population/result/#South%20America) | 88.3% | 5.84 | 3.42 | 58.59% | 2.77 | 0.97 | 95.15% | 8.61 | 4.9 |
|  |  |  |  |  |  |  |  |  |  |
| [South Asia](http://tools.iedb.org/population/result/#South%20Asia) | 94.73% | 7.27 | 4.62 | 75.38% | 3.85 | 1.62 | 98.7% | 11.12 | 7.35 |
|  |  |  |  |  |  |  |  |  |  |
| [Southeast Asia](http://tools.iedb.org/population/result/#Southeast%20Asia) | 94.56% | 7.21 | 4.57 | 56.98% | 2.68 | 0.93 | 97.66% | 9.89 | 5.88 |
|  |  |  |  |  |  |  |  |  |  |
| [Southwest Asia](http://tools.iedb.org/population/result/#Southwest%20Asia) | 92.5% | 6.91 | 4.3 | 43.93% | 1.97 | 0.71 | 95.79% | 8.88 | 5.07 |
|  |  |  |  |  |  |  |  |  |  |
| [Spain](http://tools.iedb.org/population/result/#Spain) | 87.52% | 6.07 | 3.2 | 80.51% | 4.26 | 2.05 | 97.57% | 10.32 | 6.13 |
|  |  |  |  |  |  |  |  |  |  |
| [Spain Caucasoid](http://tools.iedb.org/population/result/#Spain%20Caucasoid) | 87.52% | 6.07 | 3.2 | 80.84% | 4.28 | 2.09 | 97.61% | 10.35 | 6.16 |
|  |  |  |  |  |  |  |  |  |  |
| [Spain Other](http://tools.iedb.org/population/result/#Spain%20Other) | 0.0% | 0.0 | 0.0 | 6.3% | 0.25 | 0.43 | 6.3% | 0.25 | 0.43 |
|  |  |  |  |  |  |  |  |  |  |
| [Sri Lanka](http://tools.iedb.org/population/result/#Sri%20Lanka) | 52.39% | 2.38 | 0.84 | 0.0% | 0.0 | 0.0 | 52.39% | 2.38 | 0.84 |
|  |  |  |  |  |  |  |  |  |  |
| [Sri Lanka Asian](http://tools.iedb.org/population/result/#Sri%20Lanka%20Asian) | 52.39% | 2.38 | 0.84 | 0.0% | 0.0 | 0.0 | 52.39% | 2.38 | 0.84 |
|  |  |  |  |  |  |  |  |  |  |
| [Sudan](http://tools.iedb.org/population/result/#Sudan) | 93.78% | 7.12 | 4.48 | 60.56% | 2.84 | 1.01 | 97.55% | 9.96 | 5.93 |
|  |  |  |  |  |  |  |  |  |  |
| [Sudan Arab](http://tools.iedb.org/population/result/#Sudan%20Arab) | 70.21% | 3.7 | 1.34 | 0.0% | 0.0 | 0.0 | 70.21% | 3.7 | 1.34 |
|  |  |  |  |  |  |  |  |  |  |
| [Sudan Black](http://tools.iedb.org/population/result/#Sudan%20Black) | 2.19% | 0.09 | 0.41 | 0.0% | 0.0 | 0.0 | 2.19% | 0.09 | 0.41 |
|  |  |  |  |  |  |  |  |  |  |
| [Sudan Mixed](http://tools.iedb.org/population/result/#Sudan%20Mixed) | 94.39% | 7.37 | 4.59 | 60.56% | 2.84 | 1.01 | 97.79% | 10.21 | 6.14 |
|  |  |  |  |  |  |  |  |  |  |
| [Sweden](http://tools.iedb.org/population/result/#Sweden) | 99.99% | 12.01 | 9.34 | 88.07% | 5.02 | 3.35 | 100.0% | 17.02 | 13.28 |
|  |  |  |  |  |  |  |  |  |  |
| [Sweden Caucasoid](http://tools.iedb.org/population/result/#Sweden%20Caucasoid) | 99.99% | 12.01 | 9.34 | 88.07% | 5.02 | 3.35 | 100.0% | 17.02 | 13.28 |
|  |  |  |  |  |  |  |  |  |  |
| [Taiwan](http://tools.iedb.org/population/result/#Taiwan) | 97.77% | 8.3 | 5.36 | 67.88% | 3.34 | 1.25 | 99.29% | 11.64 | 8.12 |
|  |  |  |  |  |  |  |  |  |  |
| [Taiwan Oriental](http://tools.iedb.org/population/result/#Taiwan%20Oriental) | 97.77% | 8.3 | 5.36 | 67.88% | 3.34 | 1.25 | 99.29% | 11.64 | 8.12 |
|  |  |  |  |  |  |  |  |  |  |
| [Thailand](http://tools.iedb.org/population/result/#Thailand) | 91.21% | 6.59 | 4.13 | 63.9% | 3.09 | 1.11 | 96.83% | 9.67 | 5.56 |
|  |  |  |  |  |  |  |  |  |  |
| [Thailand Oriental](http://tools.iedb.org/population/result/#Thailand%20Oriental) | 91.21% | 6.59 | 4.13 | 63.9% | 3.09 | 1.11 | 96.83% | 9.67 | 5.56 |
|  |  |  |  |  |  |  |  |  |  |
| [Tokelau](http://tools.iedb.org/population/result/#Tokelau) | 0.0% | 0.0 | 0.0 | 55.11% | 2.42 | 0.89 | 55.11% | 2.42 | 0.89 |
|  |  |  |  |  |  |  |  |  |  |
| [Tokelau Polynesian](http://tools.iedb.org/population/result/#Tokelau%20Polynesian) | 0.0% | 0.0 | 0.0 | 55.11% | 2.42 | 0.89 | 55.11% | 2.42 | 0.89 |
|  |  |  |  |  |  |  |  |  |  |
| [Tonga](http://tools.iedb.org/population/result/#Tonga) | 0.0% | 0.0 | 0.0 | 71.91% | 3.52 | 1.42 | 71.91% | 3.52 | 1.42 |
|  |  |  |  |  |  |  |  |  |  |
| [Tonga Polynesian](http://tools.iedb.org/population/result/#Tonga%20Polynesian) | 0.0% | 0.0 | 0.0 | 71.91% | 3.52 | 1.42 | 71.91% | 3.52 | 1.42 |
|  |  |  |  |  |  |  |  |  |  |
| [Tunisia](http://tools.iedb.org/population/result/#Tunisia) | 97.52% | 8.09 | 5.28 | 74.26% | 3.77 | 1.55 | 99.36% | 11.86 | 8.31 |
|  |  |  |  |  |  |  |  |  |  |
| [Tunisia Arab](http://tools.iedb.org/population/result/#Tunisia%20Arab) | 97.52% | 8.09 | 5.28 | 74.97% | 3.82 | 1.6 | 99.38% | 11.91 | 8.34 |
|  |  |  |  |  |  |  |  |  |  |
| [Tunisia Berber](http://tools.iedb.org/population/result/#Tunisia%20Berber) | 0.0% | 0.0 | 0.0 | 74.47% | 3.77 | 1.57 | 74.47% | 3.77 | 1.57 |
|  |  |  |  |  |  |  |  |  |  |
| [Turkey](http://tools.iedb.org/population/result/#Turkey) | 44.8% | 1.79 | 0.72 | 76.19% | 3.95 | 1.68 | 86.85% | 5.74 | 3.04 |
|  |  |  |  |  |  |  |  |  |  |
| [Turkey Caucasoid](http://tools.iedb.org/population/result/#Turkey%20Caucasoid) | 44.8% | 1.79 | 0.72 | 76.19% | 3.95 | 1.68 | 86.85% | 5.74 | 3.04 |
|  |  |  |  |  |  |  |  |  |  |
| [Uganda](http://tools.iedb.org/population/result/#Uganda) | 94.87% | 7.86 | 4.74 | 0.0% | 0.0 | 0.0 | 94.87% | 7.86 | 4.74 |
|  |  |  |  |  |  |  |  |  |  |
| [Uganda Black](http://tools.iedb.org/population/result/#Uganda%20Black) | 94.87% | 7.86 | 4.74 | 0.0% | 0.0 | 0.0 | 94.87% | 7.86 | 4.74 |
|  |  |  |  |  |  |  |  |  |  |
| [Ukraine](http://tools.iedb.org/population/result/#Ukraine) | 0.0% | 0.0 | 0.0 | 50.64% | 2.19 | 0.81 | 50.64% | 2.19 | 0.81 |
|  |  |  |  |  |  |  |  |  |  |
| [Ukraine Caucasoid](http://tools.iedb.org/population/result/#Ukraine%20Caucasoid) | 0.0% | 0.0 | 0.0 | 50.64% | 2.19 | 0.81 | 50.64% | 2.19 | 0.81 |
|  |  |  |  |  |  |  |  |  |  |
| [United Arab Emirates](http://tools.iedb.org/population/result/#United%20Arab%20Emirates) | 3.37% | 0.14 | 0.41 | 32.92% | 1.32 | 0.6 | 35.19% | 1.45 | 0.62 |
|  |  |  |  |  |  |  |  |  |  |
| [United Arab Emirates Arab](http://tools.iedb.org/population/result/#United%20Arab%20Emirates%20Arab) | 3.37% | 0.14 | 0.41 | 32.92% | 1.32 | 0.6 | 35.19% | 1.45 | 0.62 |
|  |  |  |  |  |  |  |  |  |  |
| [United States](http://tools.iedb.org/population/result/#United%20States) | 99.1% | 9.86 | 6.9 | 88.1% | 5.01 | 3.36 | 99.89% | 14.87 | 10.85 |
|  |  |  |  |  |  |  |  |  |  |
| [United States Amerindian](http://tools.iedb.org/population/result/#United%20States%20Amerindian) | 99.67% | 9.13 | 6.43 | 42.79% | 1.91 | 0.7 | 99.81% | 11.03 | 8.07 |
|  |  |  |  |  |  |  |  |  |  |
| [United States Asian](http://tools.iedb.org/population/result/#United%20States%20Asian) | 97.46% | 8.63 | 5.43 | 78.84% | 4.18 | 1.89 | 99.46% | 12.81 | 8.72 |
|  |  |  |  |  |  |  |  |  |  |
| [United States Austronesian](http://tools.iedb.org/population/result/#United%20States%20Austronesian) | 0.0% | 0.0 | 0.0 | 58.09% | 2.74 | 0.95 | 58.09% | 2.74 | 0.95 |
|  |  |  |  |  |  |  |  |  |  |
| [United States Black](http://tools.iedb.org/population/result/#United%20States%20Black) | 97.11% | 8.81 | 5.46 | 71.5% | 3.61 | 1.4 | 99.18% | 12.42 | 8.38 |
|  |  |  |  |  |  |  |  |  |  |
| [United States Caucasoid](http://tools.iedb.org/population/result/#United%20States%20Caucasoid) | 99.83% | 11.43 | 8.71 | 90.15% | 5.21 | 4.01 | 99.98% | 16.64 | 12.84 |
|  |  |  |  |  |  |  |  |  |  |
| [United States Hispanic](http://tools.iedb.org/population/result/#United%20States%20Hispanic) | 98.92% | 9.34 | 6.38 | 72.95% | 3.72 | 1.48 | 99.71% | 13.06 | 9.05 |
|  |  |  |  |  |  |  |  |  |  |
| [United States Mestizo](http://tools.iedb.org/population/result/#United%20States%20Mestizo) | 99.23% | 9.46 | 6.68 | 72.23% | 3.67 | 1.44 | 99.79% | 13.13 | 9.17 |
|  |  |  |  |  |  |  |  |  |  |
| [United States Polynesian](http://tools.iedb.org/population/result/#United%20States%20Polynesian) | 99.57% | 9.86 | 7.23 | 73.18% | 3.67 | 1.49 | 99.88% | 13.52 | 9.53 |
|  |  |  |  |  |  |  |  |  |  |
| [Venezuela](http://tools.iedb.org/population/result/#Venezuela) | 90.04% | 5.29 | 4.0 | 3.01% | 0.12 | 0.41 | 90.34% | 5.41 | 4.03 |
|  |  |  |  |  |  |  |  |  |  |
| [Venezuela Amerindian](http://tools.iedb.org/population/result/#Venezuela%20Amerindian) | 90.07% | 5.24 | 4.01 | 0.0% | 0.0 | 0.0 | 90.07% | 5.24 | 4.01 |
|  |  |  |  |  |  |  |  |  |  |
| [Venezuela Caucasoid](http://tools.iedb.org/population/result/#Venezuela%20Caucasoid) | 11.45% | 0.46 | 0.45 | 0.0% | 0.0 | 0.0 | 11.45% | 0.46 | 0.45 |
|  |  |  |  |  |  |  |  |  |  |
| [Venezuela Mestizo](http://tools.iedb.org/population/result/#Venezuela%20Mestizo) | 9.75% | 0.39 | 0.44 | 0.0% | 0.0 | 0.0 | 9.75% | 0.39 | 0.44 |
|  |  |  |  |  |  |  |  |  |  |
| [Venezuela Mixed](http://tools.iedb.org/population/result/#Venezuela%20Mixed) | 0.0% | 0.0 | 0.0 | 3.17% | 0.13 | 0.41 | 3.17% | 0.13 | 0.41 |
|  |  |  |  |  |  |  |  |  |  |
| [Vietnam](http://tools.iedb.org/population/result/#Vietnam) | 91.82% | 6.66 | 4.2 | 54.44% | 2.53 | 0.88 | 96.27% | 9.19 | 5.25 |
|  |  |  |  |  |  |  |  |  |  |
| [Vietnam Oriental](http://tools.iedb.org/population/result/#Vietnam%20Oriental) | 91.82% | 6.66 | 4.2 | 54.44% | 2.53 | 0.88 | 96.27% | 9.19 | 5.25 |
|  |  |  |  |  |  |  |  |  |  |
| [Wales](http://tools.iedb.org/population/result/#Wales) | 1.0% | 0.04 | 0.4 | 0.0% | 0.0 | 0.0 | 1.0% | 0.04 | 0.4 |
|  |  |  |  |  |  |  |  |  |  |
| [Wales Caucasoid](http://tools.iedb.org/population/result/#Wales%20Caucasoid) | 1.0% | 0.04 | 0.4 | 0.0% | 0.0 | 0.0 | 1.0% | 0.04 | 0.4 |
|  |  |  |  |  |  |  |  |  |  |
| [West Africa](http://tools.iedb.org/population/result/#West%20Africa) | 95.49% | 8.04 | 4.89 | 65.23% | 3.2 | 1.15 | 98.43% | 11.24 | 7.09 |
|  |  |  |  |  |  |  |  |  |  |
| [West Indies](http://tools.iedb.org/population/result/#West%20Indies) | 98.98% | 10.0 | 7.0 | 69.22% | 3.46 | 1.3 | 99.69% | 13.46 | 9.26 |
|  |  |  |  |  |  |  |  |  |  |
| [World](http://tools.iedb.org/population/result/#World) | 98.55% | 9.19 | 6.06 | 81.81% | 4.43 | 2.2 | 99.74% | 13.62 | 9.43 |
|  |  |  |  |  |  |  |  |  |  |
| [Zambia](http://tools.iedb.org/population/result/#Zambia) | 98.1% | 8.57 | 5.55 | 0.0% | 0.0 | 0.0 | 98.1% | 8.57 | 5.55 |
|  |  |  |  |  |  |  |  |  |  |
| [Zambia Black](http://tools.iedb.org/population/result/#Zambia%20Black) | 98.1% | 8.57 | 5.55 | 0.0% | 0.0 | 0.0 | 98.1% | 8.57 | 5.55 |
|  |  |  |  |  |  |  |  |  |  |
| [Zimbabwe](http://tools.iedb.org/population/result/#Zimbabwe) | 93.79% | 7.24 | 4.49 | 68.3% | 3.35 | 1.26 | 98.03% | 10.6 | 6.45 |
|  |  |  |  |  |  |  |  |  |  |
| [Zimbabwe Black](http://tools.iedb.org/population/result/#Zimbabwe%20Black) | 93.79% | 7.24 | 4.49 | 68.3% | 3.35 | 1.26 | 98.03% | 10.6 | 6.45 |
|  |  |  |  |  |  |  |  |  |  |
| **Average** | **63.06** | **5.34** | **3.61** | **55.3** | **2.81** | **1.39** | **82.92** | **8.15** | **5.36** |
| **Standard deviation** | **43.68** | **4.17** | **3.03** | **30.26** | **1.68** | **1.11** | **25.25** | **4.78** | **3.8** |
